# Small nucleolar RNA dysregulation and potential roles in bovine subclinical mastitis

**DOI:** 10.1186/s40104-025-01230-9

**Published:** 2025-09-12

**Authors:** Faith A. Omonijo, Mengqi Wang, David Gagné, Mario Laterrière, Samuel Genier, Xin Zhao, Eveline M. Ibeagha-Awemu

**Affiliations:** 1 Sherbrooke Research and Development Centre, Agriculture and Agri-Food Canada, Sherbrooke, QC Canada; 2 Department of Animal Science, McGill University, Ste-Anne-De-Bellevue, QC Canada; 3 Quebec Research and Development Centre, Agriculture and Agri-Food Canada, Quebec, QC Canada

**Keywords:** Dairy cattle, Ribosomal RNA, *S. aureus*, *S. chromogenes*, SnoRNA, Subclinical mastitis

## Abstract

**Background:**

Subclinical mastitis, caused by many pathogens including *Staphylococcus aureus* (*S. aureus*) and *Staphylococcus chromogenes (S. chromogenes)*, presents a major challenge to the dairy industry due to its associated economic losses and poor milk quality. The molecular regulatory mechanisms, including the role of small nucleolar RNAs (snoRNAs), of the host response to mastitis pathogens remain unclear. Therefore, this study investigated snoRNA expression and potential roles during subclinical mastitis. Milk somatic cells from cows with naturally occurring *S. aureus* (*n* = 14) and *S. chromogenes* (*n* = 3) subclinical mastitis, and healthy cows (*n* = 4) were subjected to transcriptome sequencing and bioinformatics analyses.

**Results:**

We identified 255 expressed snoRNAs including 21 differentially expressed (DE) in *S. aureus-*positive cows and 20 DE in *S. chromogenes*-positive cows. Prediction of ribosomal RNA (rRNA) modification sites found several 18S rRNA and 28S rRNA modification (pseudouridylation and 2′-O-methylation) target sites essential for ribosome function for DE snoRNAs, such as SNORA79 (18S-1319, 28S-3001), SNORA1 (18S-1496, 28S-1747), suggesting their roles in translation and immune modulation during subclinical mastitis. Correlation analysis identified DE snoRNAs-mRNAs (from the same samples) pairs with majority of the correlated mRNAs (e.g., *CXCL8*,* IL6R*,* IL2*,* IL1R*,* IL18R1*,* STAT3*,* NFKB2*,* MYD88*,* VEGFA*,* and CD40*) having immune related functions. Functional enrichment of correlated genes of snoRNAs for *S. aureus-*positive group (regulation of defense/immune response, leukocyte differentiation, response to cytokine, NF-κB signaling pathway, JAK-STAT signaling pathway etc.) and *S. chromogenes-*positive group (e.g., regulation of defense response, response to cytokine, regulation of immune response, NF-κB signaling pathway, TNF signaling pathway, and JAK-STAT signaling pathway) revealed involvement in immune and inflammatory processes. Some functional terms were common to both pathogens (e.g., NF-κB, JAK-STAT signaling, immune system processes) and suggest common regulatory mechanisms used by both pathogens to contain infection. Furthermore, snoRNA-mRNA network construction identified 7 key (hub) snoRNAs each for *S. aureus*-positive group (SNORA66, novelsnoRNA_26_14905 (also denoted as novelSnoRNA_86), SNORD107, SNORA1, SNORA63, SNORA79, SNORA76) and *S. chromogenes-*positive group (SNORD18, SNORA79, SNORA46, U2-19, SNORA66, SNORD37, SNORD49) that correlated with the most protein coding genes (|r| > 0.9; ≥ 30 mRNAs). Functional enrichment of correlated genes of hub snoRNAs reveals their involvement in immune related functions (75% of enriched terms) and metabolic processes (20% of enriched terms).

**Conclusion:**

These data suggest potential regulatory roles for the DE snoRNAs and in particular, the 14 hub snoRNAs during subclinical mastitis. This study presents the first evidence linking snoRNAs to bovine subclinical mastitis and offers new insights into the molecular mechanisms underlying subclinical mastitis caused by *S. aureus* and *S. chromogenes*.

**Supplementary Information:**

The online version contains supplementary material available at 10.1186/s40104-025-01230-9.

## Introduction

Mastitis, characterized by inflammation of the mammary gland, is the most prevalent and costly disease in dairy livestock globally [1]. Depending on the causal pathogen and degree of inflammation, mastitis can develop as a clinical, or subclinical infection. Subclinical mastitis caused by various pathogens, including *Staphylococcus aureus* (*S. aureus*) and *Staphylococcus chromogens* (*S. chromogenes*), lacks visible symptoms, making it difficult to detect, thus leading to substantial economic losses. *Staphylococcus aureus* as the most important mastitis pathogen has the ability to evade host immune cells, continually multiply within the host cells, and thereby causing frequent infections. On the other hand, in dairy production, *S. chromogens* is often considered a minor pathogen [2, 3]. Despite this, *S. chromogenes* is frequently isolated from cows with subclinical and clinical mastitis [4] and it is the most prevalent non-aureus staphylococci (NAS) in Canadian mastitis cases regardless of somatic cell count (SCC) levels [5]. Similar to *S. aureus*, *S. chromogenes* can penetrate the teat canal, attach to cells, and form biofilms, preventing removal during milking [4]. Thus, like *S. aureus, S. chromogenes* is becoming more prevalent and problematic.

High rate of *S. chromogenes* intra-mammary infection in udder quarters with high SCCs, persistent cases of subclinical mastitis, and more pronounced signs of clinical mastitis has been reported [4]. Given the high prevalence of subclinical mastitis and its impact on dairy production, including the devastating impacts of the development of antimicrobial/antibiotic resistance on animal, human, and environmental health, it is crucial to deepen our understanding of the molecular regulation and genetic basis of mastitis. This knowledge is essential for developing effective strategies for managing udder health, such as development of therapeutic solutions and genomic selection for mastitis resistance.

The epigenome including non-coding RNA (ncRNA) regulation, plays a crucial role in regulating gene expression and has been shown to regulate various livestock traits, including mammary gland health and production [6–9]. In particular, crucial roles of ncRNAs, including microRNAs (miRNAs), long ncRNAs (lncRNAs) and circular RNAs (circRNAs) have been reported for animal diseases [7, 10–12]. Among these, miRNAs and lncRNAs are the most extensively studied, while others like small nucleolar RNAs (snoRNAs) have received less attention despite being comparable to miRNAs and lncRNAs in abundance.

SnoRNAs, primarily located in the eukaryotic cell nuclei, are 60–300 nucleotides long and contain conserved structural elements. They are classified into two main types based on their structural components and biological roles: H/ACA snoRNAs (SNORA), which guide the pseudouridylation of nucleotides, and C/D box snoRNAs (SNORD), which are responsible for 2'-O-methylation. Recent data suggest that snoRNAs also modify small nuclear RNAs, transfer RNAs, and even messenger RNAs, and are associated with the pathogenesis of various malignancies, indicating their potential as prognostic biomarkers [13]. Studies have also confirmed that snoRNAs can perform miRNA-like functions by regulating post-transcriptional gene expression [14, 15]. In 2008, Ender and colleagues reported small RNAs derived from snoRNA ACA45 with miRNA-like functions by targeting *CDC2L6* [16]. Also, Ono et al. [15] showed that while the C/D box snoRNA HBII-180C contains a 2′-O-methylation site, it also has an M-box region that allows it to function as a miRNA, inhibiting the mRNA and protein expression of target genes [15]. Huang et al. [17] and Asano-Inami et al. [18] found that SNORD50A and SNORD49 inhibited mRNA 3' processing by blocking the interaction between *Fip1* (a component of cleavage and polyadenylation specificity factor) and the polyA site. This inhibition led to alterations in alternative polyadenylation (APA) profiles and transcript levels of various genes. Decreased expression of snoRNAs, including SNORD49, reduces the interaction between *UBAP2L* and *G3BP1,* thereby suppressing stress granules formation [18]. McCann et al. revealed that SNORA1 was significantly downregulated during stem cell differentiation, suggesting a potential role in the differentiation process [19]. The regulation of SNORA1 and other H/ACA snoRNAs may be crucial for proper cell differentiation, as these snoRNAs are involved in the modification and processing of rRNA, which is essential for ribosome biogenesis and overall cellular function. Taken together, these data highlight the importance of snoRNAs in cellular differentiation and their potential implications in developmental biology and disease states. Despite the demonstrated functions of snoRNAs in human diseases, their regulatory roles in livestock diseases including mastitis are yet to be elucidated. This study therefore aims to characterize snoRNA expression and their potential functions during bovine subclinical mastitis. To the best of our understanding, this is the first study to characterize snoRNA expression and their potential regulatory roles in bovine *S. chromogenes* and *S. aureus* subclinical mastitis.

## Materials and methods

### Ethics approval and consent for participation

The animal use procedures for this study adhered to the Canadian Council on Animal Care guidelines. Ethical approval was granted by the Animal Care and Ethics Committee of Agriculture and Agri-Food Canada (CIPA #570).

### Farms and animal selection

Five commercial dairy farms in Quebec operating the tie-stall management system were selected for this study. Monthly dairy herd improvement (DHI) records (milk production) of lactating cows were monitored over a period of six months. The DHI records generated by Lactanet (www.lactanet.ca) were based on the analysis of monthly milk samples collected from each cow in the participating herds and included fat percentage (%), protein%, lactose, milk urea nitrogen and milk somatic cell counts (SCC) amongst others. Cows with consistently very high milk SCC (an indirect measure of mammary gland health) (> 350,000 cells/mL) or consistently very low milk SCC (< 100,000 cells/mL) for three consecutive months were recruited for the study.

### Pathogen identification and milk sampling

The procedures for pathogen identification and milk sampling have been described previously [20]. In brief, to test for the presence of pathogens, 10 mL of milk samples were aseptically collected from each quarter of cows in the high SCC group or a composite milk sample (equal volumes from all four quarters) per cow in the low SCC group. The samples were kept on ice and sent to Biovet laboratories (https://www.biovet-inc.com/) on the same day for bacteriological analysis. The analysis included testing for mastitis-causing microorganisms such as non-fastidious bacteria and mesophilic microbes (e.g., *Staphylococcus* species, *Escherichia* species, *Streptococcus* species, *Klebsiella* species, *Nocardia* species, *Aerococcus* species, *Micrococcus* species, yeast species and many others). Fourteen cows from the high SCC group that tested positive to *S. aureus* only were selected to constitute the *S. aureus*-positive group or three cows positive to *S. chromogenes* only constituted the *S. chromogenes-*positive group. Four low SCC cows that tested negative for all mastitis pathogens tested served as the healthy control (HC) group. Cows that were positive to other pathogens or to multiple pathogens were excluded from the study. Subsequently, 200 mL of milk was collected from one positive quarter (even if multiple quarters were infected) of each cow in the *S. aureus*-positive and *S. chromogenes*-positive groups. For the HC group, a 200-mL composite sample (50 mL from each quarter) was collected from each cow. A second bacteriological test on 10 mL of milk from these samples was performed to confirm the initial results. Only samples with consistent findings from both tests were used for further analysis. Milk samples were immediately transported on ice to the laboratory for somatic cells isolation. Milk somatic cells were isolated by low speed centrifugation (1,500 × *g* for 15 min at 4 °C) followed by removal of the fat and whey layers. The somatic cells were then washed twice with phosphate buffered saline (PBS) by adding 40 mL of 1 × PBS and centrifuging at 1,500 × *g* for 15 min at 4 °C. Finally, the washed somatic cells were treated with TRIzol reagent (Qiagen Inc, Toronto, ON, Canada) and stored at −80 °C for further use.

### RNA isolation and quality control

Total RNA was extracted from milk somatic cells with RNeasy Mini Kit (Qiagen Inc., Toronto, ON, Canada) according to the manufacturer’s protocol. Agilent Bioanalyzed 2100 (Agilent Technologies, Saint-Laurent, QC, Canada) was used to quantify RNA, and LabChip GXII (PerkinElmer Inc., Waltham, MA, USA) was used to assess RNA integrity. RNA samples with RIN (RNA integrity number) values above 7 were processed further.

### Library preparation and sequencing

Ribosomal RNA was depleted from 125 ng of total RNA using the QIAseq FastSelect Kit (Qiagen Inc., Toronto, ON, Canada). Complementary DNA was synthesized using the NEBNext RNA First Strand Synthesis and NEBNext Ultra Directional RNA Second Strand Synthesis Modules (New England BioLabs, Whitby, ON, Canada). Library preparation was performed with the NEBNext Ultra II DNA Library Prep Kit for Illumina (New England BioLabs, Whitby, ON, Canada). Adapters and polymerase chain reaction primers were purchased from New England BioLabs. Library quantification was performed with the Kapa Illumina GA with Revised Primers-SYBR Fast Universal kit (Kapa Biosystems, Wilmington, MA, USA). The average fragment size was assessed using the LabChip GXII instrument (PerkinElmer, Woodbridge, ON, Canada).

The libraries were normalized and pooled (equimolar concentrations) and then denatured in 0.05 mol/L NaOH and neutralized using HT1 buffer. The pool was loaded at 200 pM on an Illumina NovaSeq 6000 lane using Xp protocol as per the manufacturer’s recommendations. The run was performed for 2 × 100 cycles (paired-end mode). A phiX library was used as a control and mixed with libraries at 1% level. Base calling was performed with Real-Time Analysis (version 3.4.4). Bcl2fastq2 v2.20 program was used to demultiplex samples and generate fastq reads. Sequencing was performed by Centre d'expertise et de services Génome Quebec (https://www.genomequebec.com/).

### Bioinformatics analyses

**Fig. 1:**
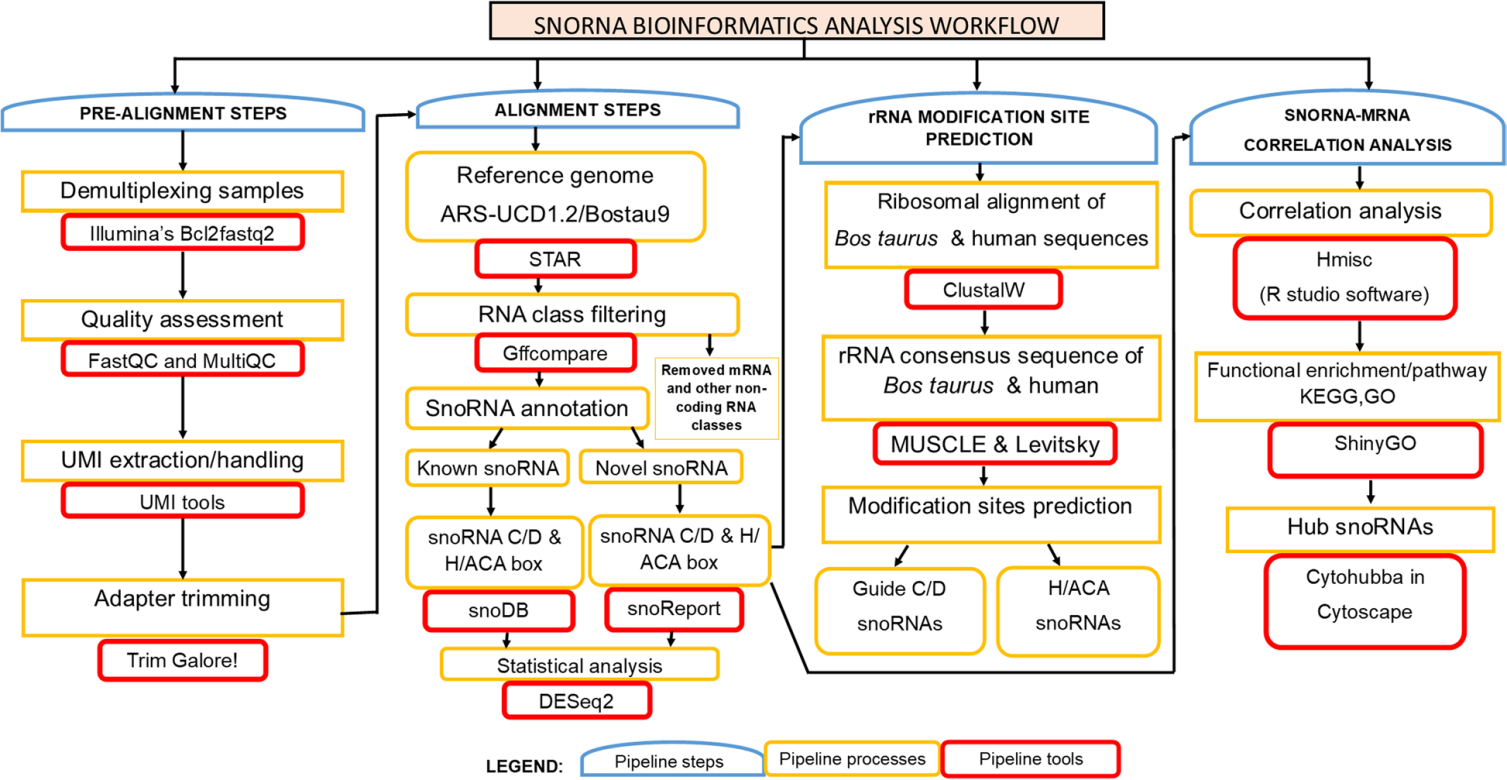
Flowchart of small nucleolar RNA bioinformatics analysis

RNA sequencing data was processed using nf-core/rnaseq analysis pipeline version 3.3 [21]. The steps in bioinformatics analysis are summarized in Fig. 1. Quality assessment of the sequences was performed with FastQC (version 0.11.9). Barcodes were removed with UMI-tools (https://github.com/CGATOxford/UMI-tools). Three prime adaptor sequences and 5′ adaptor contaminants and repeats were trimmed using Trim Galore! (version 0.6.5). Likewise, low-quality reads (i.e. reads shorter than 60 nucleotides or having a low Phred score of less than 20 for at least 50% of the bases) were removed. The high-quality trimmed reads were merged and mapped to the bovine reference genome (ARS-UCD1.2/Bostau9 genome) using STAR (https://github.com/alexdobin/STAR). Then reads of mRNAs and other classes of non-coding RNAs were removed. The General Feature Format (GFF) file was downloaded from the ARS-UCD1.2/Bostau9 genome database. The GFF file was modified to retain only entries corresponding to known snoRNAs by applying the grep command as follows: “grep snoRNA ARS-UCD1.2_RefSeq_other_rna.gff3 > ARS-UCD1.2_RefSeq_snoRNA.gff”. Subsequent modifications to the pipeline were implemented using Nextflow, particularly adjusting the trim_galore and feature counts modules for compatibility with the data. Box C/D and H/ACA box snoRNAs of the known snoRNAs were identified in the trimmed and filtered reads using snoDB (https://bioinfo-scottgroup.med.usherbrooke.ca/snoDB/) [22]. The remaining reads after annotating the known snoRNAs were used in identifying the novel snoRNAs using snoReport (version 2.0; https://joaovicers.github.io/snoreport2/), a tool that identifies box C/D and H/ACA box snoRNAs, using a combination of secondary structure prediction and machine learning [23]. The analysis of snoRNA expression differences between *S. chromogenes-*positive or *S. aureus-*positive group, and HC groups were conducted using DESeq2 (version 3.15) [24]. Lactation stage and treatment were included as batch factors for cows in the *S. chromogenes-*positive group while lactation stage, parity and treatment were included as batch factors for cows in the *S. aureus-*positive group during analysis. Statistically significant differentially expressed (DE) snoRNAs were defined as having a Benjamini and Hochberg [25] corrected false discovery rate (FDR) < 0.05 and log_2_ fold change (log_2_FC) > 1. Scripts used for the snoRNA analyses are available at https://github.com/EIAlab/snoRNA.

### snoRNA-rRNA modification site prediction

Ribosomal RNA modification site prediction of snoRNAs in *S. aureus-*positive and *S. chromogenes-*positive samples were determined with snoRNA target prediction tools as described previously [26, 27] and shown in Fig. 1. Briefly, ribosomal alignments of *Bos taurus* and human ribosomal sequences by type (5.8S, 18S and 28S) was performed using ClustalW embedded in the MEGA 11 software in Ubuntu 22.04 LTS (https://www.megasoftware.net/). To predict the modification sites in the ribosomal RNA sequence, consensus sequences were created by aligning 18S_Human (U13369.1:3657–5527) and 18S_Btaurus (NR_036642.1), 28S_Human (U13369.1:7935–12969) and 28S_Btaurus (DQ222453.1:4134–8675), and 5.8S_Human (U13369:6623–6779) and 5.8S_Btaurus (DQ222453.1:2946–3095) with UGENE software (v45.1, 64-bit version). MUSCLE algorithm and Levitsky consensus type were selected for the alignment. The consensus RNA sequence was used in RNAsnoop (version 2.5.1 [28]) and snoScan (version 1.0) [29] to predict the methylation site of H/ACA box (2′-O-ribose methylation; Nm) and guide C/D box (pseudouridylation; Ψ) snoRNA, respectively, using the default parameters. Also, snoDB (https://bioinfo-scottgroup.med.usherbrooke.ca/snoDB/), an up-to-date interactive database of human snoRNAs used to predict the function of the H/ACA box and C/D box snoRNAs, were used to further confirm the methylation sites of H/ACA box and C/D box snoRNAs in the studied samples.

### Construction of snoRNA-mRNA co-expression networks and functional annotation of co-expressed genes

SnoRNA functions to regulate mRNA gene expression at the post transcriptional level. Therefore, Pearson correlation of the expression values of DE snoRNAs from *S. aureus-*positive and *S. chromogenes-*positive groups and DE mRNAs of the same groups [30] was conducted to identify DE mRNAs correlating positively or negatively with DE snoRNAs using Hmisc, a R studio software (Version 5.1.3), with the threshold of *P* < 0.05 and |*r*| > 0.9. The analysis of the mRNA expression from the same samples and in relation to the studied pathogens has been reported previously [30]. The mRNAs that correlated positively or negatively with snoRNAs were subjected to functional analysis to get further insights into the functions of snoRNAs. Functional enrichment (KEGG pathway and Gene Ontology (GO)) analysis was performed with ShinyGO (version 0.80) (http://bioinformatics.sdstate.edu/go/). Annotation terms with FDR < 0.05 were regarded as statistically significant and the correlation networks of snoRNA-mRNA were visualized with cytoscape software (version 3.9.1) (http://www.cytoscape.org/) [31]. The top 7 snoRNAs (according to degree values) that correlated with the most mRNAs were selected as hub snoRNAs using cytohubba (version 3.0) [32], a cytoscape plug in for the *S. aureus*-positive and *S. chromogenes*-positive groups. Functional enrichment analysis was performed for the DE mRNAs that correlated with the hub snoRNAs. Cytoscape was used to construct hub snoRNA-mRNA interaction network.

### Real-time qPCR validation of snoRNA expression

The expression levels of four randomly selected snoRNA genes, comprising 3 DE and 1 non-DE snoRNAs, were analyzed using real-time qPCR to validate RNA-sequencing data (Supplemental Table S7). Primers for the genes were designed with Primer-BLAST (https://www.ncbi.nlm.nih.gov/tools/primer-blast/). For cDNA synthesis, 200 ng of total RNA per sample was used. The total RNA was extracted from blood samples (stored in PAXgene tubes, Qiagen Inc, Toronto, ON, Canada) from the same *S. aureus* positive cows and healthy cows used in snoRNA analysis. Take note that blood samples from the same animals were used for this validation because the RNA isolated from milk somatic cells were no longer available (finished). The total RNA was reverse transcribed using the SuperScript™ IV VILO™ Master Mix (Invitrogen, Waltham, Massachusetts, USA). The resulting cDNA was diluted 1:10 and used for gene-specific qPCR amplification. The 20 µL qPCR reaction mixture included 2 µL (10 ng) of cDNA, 5 µL Power SYBR^®^ Green PCR Master Mix (Applied Biosystems, Waltham, Massachusetts, USA), 1 µL each of forward and reverse primers (500 nmol/L each), and 7 µL nuclease-free water. Real-time amplification was performed on a StepOnePlus™ instrument (Applied Biosystems, Waltham, Massachusetts, USA) using a standard cycling mode. The cycling protocol began with a 10 min enzyme activation at 95 °C, followed by 40 cycles of denaturation at 95 °C for 15 s and extension at 60 °C for 60 s. *RPS9* was used as the reference gene to normalize gene expression levels, and the relative expression values were calculated using the 2^−∆∆Ct^ method [33].

## Results

### SnoRNA expression profiles in milk somatic cells during subclinical mastitis

High-throughput RNA sequencing produced an average of 23.32 million, 16.68 million and 18.14 million raw reads per sample for *S. aureus-*positive, *S. chromogenes-*positive and HC groups, respectively (Supplemental Table S1). After removing adaptor and low-quality sequences, *S. aureus-*positive, *S. chromogenes-*positive and HC groups yielded an average of 17.25, 12.9 and 14.22 million clean reads (75%) per sample, respectively. The average GC content per sample was approximately 0.44% for each of the three groups. The clean reads were mapped to the bovine reference genome (ARS-UCD1.2). Uniquely mapped reads comprised 92.42% of the total clean reads (Supplemental Table S1). Principal component analysis (PCA) was conducted to assess the overall relationship between samples. The PCA plot revealed that most samples clustered into two main groups: the *S. aureus-*positive group and HC group (Supplemental Fig. S1 A) and the *S. chromogenes-*positive group and HC group (Supplemental Fig. S1B). The differences between *S. chromogenes*-positive and HC groups accounted for 36% of the variance, while the differences within groups accounted for 25% of the variance. Likewise, the differences between *S. aureus-*positive and HC groups accounted for 40% of the variance, while the differences within groups accounted for 15% of the variance demonstrating the reliability of the sample phenotypes for further analysis.

### Differential snoRNA expression in response to *S. aureus* and *S. chromogenes* infection

**Fig. 2:**
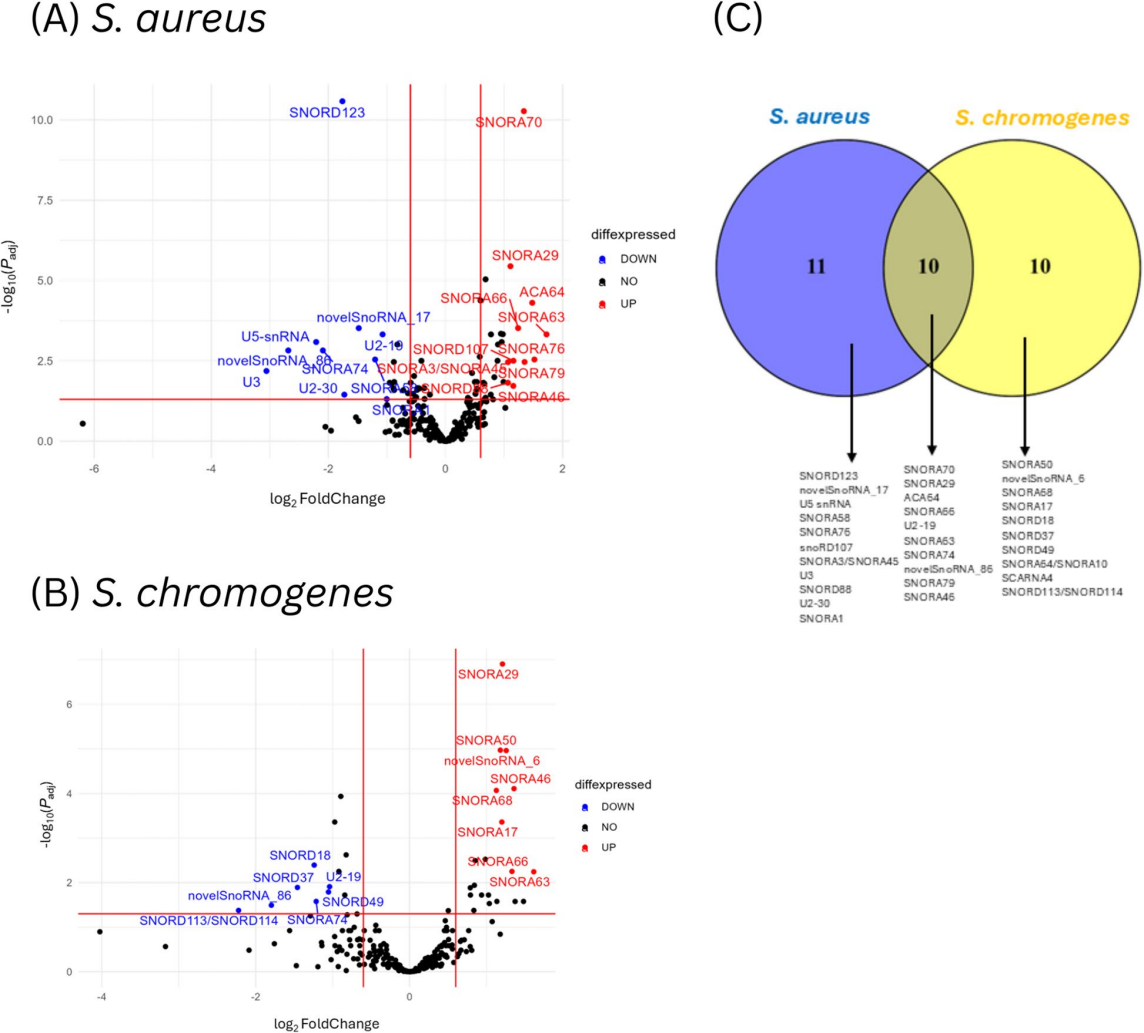
Differentially expressed (DE) snoRNAs identified in *S. aureus*-positive and *S. chromogenes*-positive groups. **A** Volcano plot showing DE snoRNAs between *S. aureus*-positive vs. control groups. **B** Volcano plot showing DE snoRNAs between *S. chromogenes*-positive vs. control groups. **C** Venn diagram showing the total number of DE snoRNAs that are specific and common to the two pathogens (*S. aureus* and *S. chromogenes*)

A total of 255 snoRNAs were identified in the samples after filtering out reads with fewer than 10 read counts and reads mapping to mRNA and other ncRNA species. Among these, 233 were known snoRNAs and 20 were novel (Supplemental Table S2). The expression levels of snoRNAs were compared between the *S. aureus-*positive and HC groups, and between *S. chromogenes-*positive and HC groups to identify DE snoRNAs. A total of 21 snoRNAs (18 known and 3 novel) exhibited significant differential expression between the *S. aureus-*positive group and HC group (FDR < 0.05 and log_2_FC > 1; Supplemental Table S3A). Among the *S. aureus-*positive group DE snoRNAs, 11 were upregulated, and 10 were downregulated (Fig. 2A; Supplemental Table S3A). Several known snoRNAs including SNORD88, SNORA76, SNORA29, SNORA46, snoRD107, SNORA66, SNORA70, SNORA79, ACA64, SNORA76 and SNORA63 were significantly upregulated in the *S. aureus-*positive group (Fig. 2A; Supplemental Table S3A). In contrast, many known snoRNAs including U3, SNORA74, SNORD123, U2-30, SNORA58, U2-19, SNORA1 and three novel snoRNAs (novelsnoRNA_26_14905, novelsnoRNA_4_112140344 and novelsnoRNA_10_7030513) were significantly down-regulated in the *S. aureus-*positive group compared to the HC group (Fig. 2A; Supplemental Table S3B).

Comparing the *S. chromogenes-*positive group and HC group, 20 snoRNAs were significantly DE (FDR < 0.05 and log_2_FC > 1; Supplemental Table S3A). Among the *S. chromogenes-*positive group DE snoRNAs, 13 were upregulated and 7 were downregulated (Fig. 2B; Supplemental Table S3B). Several snoRNAs including SNORA64/SNORA10, SNORA70, SNORA68, SNORA79, SNORA50, SNORA17, SNORA29, novelsnoRNA_2_26696606, SNORA66, SNORA46, SCARNA4, ACA64 and SNORA63) were significantly up-regulated (Fig. 2B) while SNORD113/SNORD114, novelsnoRNA_26_14905, SNORD37, SNORD18, SNORA74, SNORD49 and U2-19 were significantly down-regulated (Fig. 2B) in the *S. chromogenes*-positive group. Additionally, ten snoRNAs (SNORA74, novelsnoRNA_26_14905, SNORA29, SNORA46, SNORA66, SNORA70, SNORA79, ACA64, U2-19 and SNORA63) were present in both *S. aureus-*positive group and *S. chromogenes-*positive group (Fig. 2C), suggesting these snoRNAs may play similar roles in the regulation of mastitis induced by both pathogens. Meanwhile 11 snoRNAs (U3, U5 snRNA, SNORD123, U2-30, novelsnoRNA_4_112140344, SNORA58, SNORA1, SNORD88, SNORD107, SNORA76 and SNORA3/SNORA45) were DE in *S. aureus-*positive group only and 10 snoRNAs (SNORD113/SNORD114, SNORD37, SNORD18, SNORD49, SNORA64/SNORA10, SNORA68, SNORA50, SNORA17, novelsnoRNA_2_26696606 and SCARNA4) were DE in *S. chromogenes-*positive group only (Fig. 2C). These findings suggest that the DE snoRNAs unique to each pathogen could regulate processes triggered in response to the degree of pathogenicity of each pathogen. Meanwhile, DE snoRNAs shared between the two pathogens highlight the important role of snoRNAs as key regulators of inflammatory responses caused by *S. chromogenes* and *S. aureus*.

### Prediction of rRNA 2′-O-methylation and pseudouridylation binding sites on small nucleolar RNA

To understand the roles of snoRNAs in *S. aureus* and *S. chromogenes* subclinical mastitis, we identified the potential modification sites of rRNAs 2′-O-methylation and pseudouridylation binding sites on the DE snoRNAs. We discovered that from the 21 DE snoRNAs found in *S. aureus-*positive group (7 from the C/D subfamily, 13 from the H/ACA subfamily and 1 from snRNA subfamily), 17 of the DE snoRNAs could target 34 specific modification sites on rRNAs (14 modification sites on 18S rRNA and 20 modification sites on 28S rRNA; Table 1) while from the 20 DE snoRNAs found in *S. chromogenes-*positive group (6 from the C/D subfamily, and 14 from the H/ACA subfamily), 14 could target 29 specific modification sites on rRNAs (13 modification sites on 18S rRNA and 16 modification sites on 28S rRNAs; Table 2). Notably for *S. aureus-*positive group, three snoRNA C/D box family members were predicted to direct 2′-O-methylation at Um1911, Um3320, Cm451 Um3320 (SNORD123), Um4158 (SNORD107) and Am1434 (U3) on 28S rRNA (Table 1), while two snoRNAs are predicted to direct 2′-O-methylation at Am1200 and Am3289 (SNORD18), and 3261 (SNORD37) for the *S. chromogenes-*positive group (Table 2). The remaining 19 snoRNAs from *S. aureus-*positive group and 18 snoRNAs from *S. chromogenes-*positive group, are predicted to modify pseudouridylation sites on 18S and 28S rRNAs (Tables 1 and 2). For *S. aureus* and *S. chromogenes* groups, SnoDB results showed that SNORA66, SNORA63, SNORA1, SNORA3, SNORA79, SNORA46 and SNORD37 were involved in ribosome biogenesis, processing, transcription and translation regulation (Supplemental Table S4A and S4B). These results suggest that snoRNA could be potentially involved in regulating ribosomal RNA processing and translation during bovine subclinical mastitis.

**Table 1:** Target modification sites for 17 snoRNAs binding found on 18S and 28S rRNAs for *S. aureus*-positive group

snoRNA_ID	snoRNA box types	Target modification sites	
		18S rRNA (2′-O-methylation site)	28S rRNA (2′-O-methylation site)
SNORD123	C/D Box	-	Um1911, Um3320, Cm451, Um3320
snoRD107	C/D Box	-	Um4158
U3	C/D Box	-	Am1434
		**18S rRNA (Pseudouridylation site)**	**28S rRNA (Pseudouridylation site)**
SNORA70	H/ACA	1323	3011
SNORA29	H/ACA	1329	4262
ACA64	H/ACA	1333	267
SNORA66	H/ACA	1323	4232
U2-19	H/ACA	1359	2309
SNORA63	H/ACA	688	3007
SNORA74	H/ACA	596	263
SNORA58	H/ACA	1608	2930
SNORA76	H/ACA	1323	3534
SNORA79	H/ACA	1319	3001
SNORA76	H/ACA	1667	3463
SNORA46	H/ACA	1319	941
SNORA1	H/ACA	1496	1747
U5 snRNA	snRNA	1350	3617

**Table 2:** Target modification sites for 14 snoRNAs binding found on 18S and 28S rRNAs for *S. chromogenes-*positive group

snoRNA_ID	snoRNA box types	Target modification sites	
		18S rRNA (2′-O-methylation site)	28S rRNA (2′-O-methylation site)
SNORD18	C/D Box	-	Am1200
SNORD37	C/D Box	-	Am3289, 3261
		**18S rRNA (Pseudouridylation site)**	**28S rRNA (Pseudouridylation site)**
SNORA29	H/ACA	1329	4262
SNORA50	H/ACA	1155	2954
SNORA46	H/ACA	1154	941
SNORA6	H/ACA	443	273
SNORA17	H/ACA	945	239
SNORA66	H/ACA	1323	4232
SNORA63	H/ACA	1340, 688	239, 3007
SNORA64/SNORA10	H/ACA	954	579
SNORA74	H/ACA	596	263
SNORA79	H/ACA	1319	3001
ACA64	H/ACA	1333	267
SNORA70	H/ACA	1323	3011

### Identification of snoRNAs co-expressed protein-coding genes and functional enrichment

**Fig. 3:**
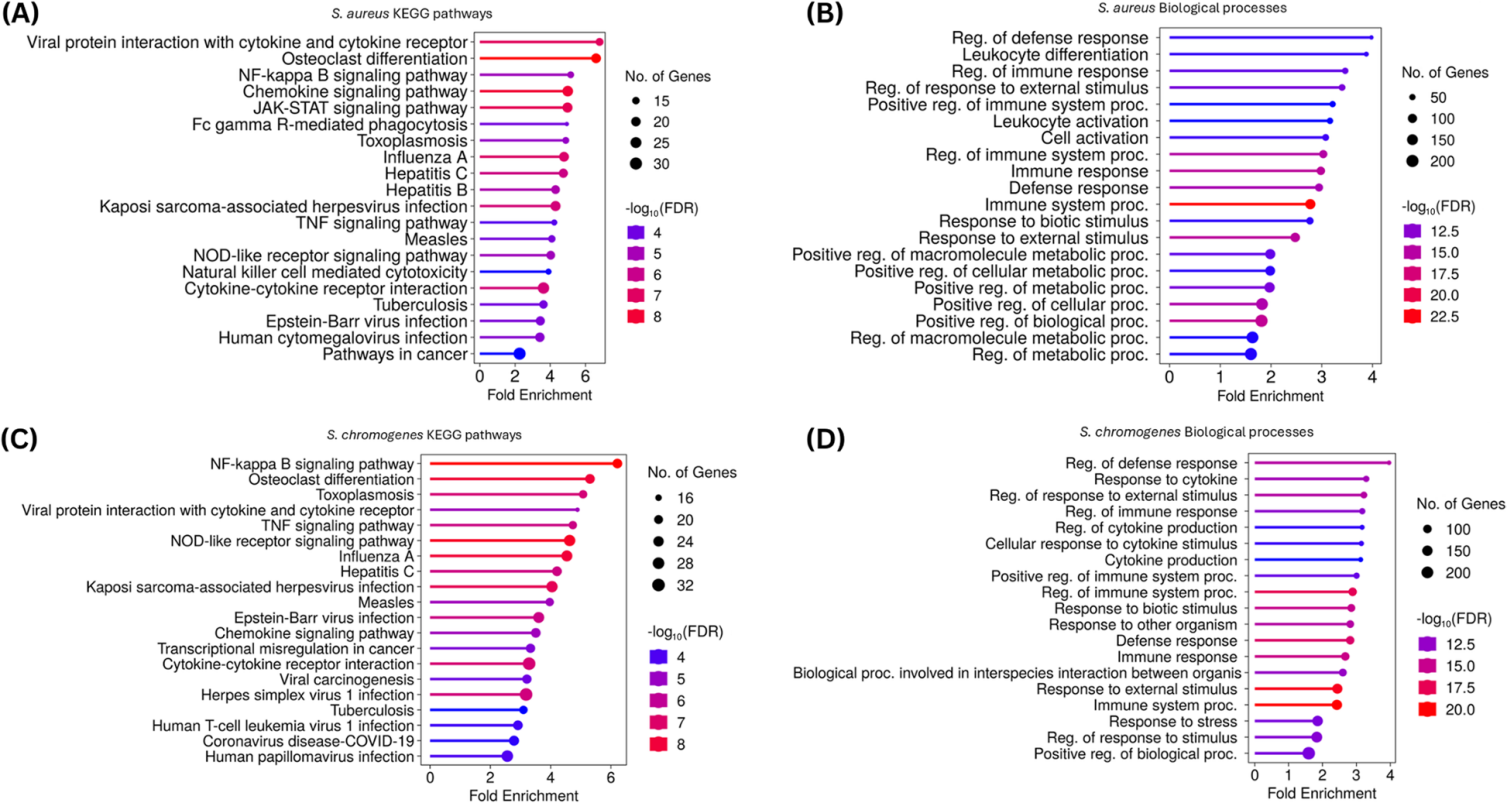
Functional pathway annotation and Gene Ontology Biological Processes terms of DE snoRNA correlated genes from *S. aureus* and *S. chromogenes-*positive groups. **A** The top 20 KEGG pathways with the largest gene ratios are plotted in order of gene ratio for *S. aureus* **B**. The top 20 GO biological processes terms with the largest gene ratios are plotted in order of gene ratio for *S. aureus*. **C** The top 20 KEGG pathways with the largest gene ratios are plotted in order of gene ratio for *S. chromogenes*. **D** The top 20 GO biological processes terms with the largest gene ratios are plotted in order of gene ratio for *S. chromogenes*. The size of the dots represent the number of genes in the significant DE gene list associated with the pathway and GO term and the color of the dots represent the *P*-adjusted values (FDR < 0.05)

SnoRNAs and their associated mRNAs have been implicated in post-transcriptional processes that contribute to disease development. To investigate these relationships, we performed a correlation analysis to identify snoRNAs and their negatively and positively co-expressed protein-coding genes. Co-expressed mRNAs with correlation coefficients > 0.9 were retained for further functional analysis. A total of 3,550 co-expressed genes for the 21 DE snoRNAs from the *S. aureus-*positive vs. HC comparison (Supplemental Table S5A) and 1,316 co-expressed genes for the 20 DE snoRNAs from the *S. chromogenes-*positive group vs. HC comparison (Supplemental Table S5B), were identified. To further understand the function of the DE snoRNAs, KEGG and GO enrichment analyses were performed using SHINYGO. The *S. aureus-*positive group correlated genes were significantly enriched in 85 KEGG pathways (FDR < 0.05; Supplemental Table S6A) and 1,000 biological process (BP) GO terms (FDR < 0.05, Supplemental Table S6B). Among the top 20 KEGG pathways in the *S. aureus-*positive group are immune and disease pathways such as NF-κB signaling pathway, Chemokine signaling, JAK-STAT signaling, Cancer pathways, and TNF signaling, among others (Fig. 3A). Consistent with the KEGG pathways, the top 20 significant GO-BP terms involved in immune responses included regulation of defense response, leukocyte differentiation, and regulation of immune response, among others (Fig. 3B). In the *S. chromogenes*-positive group, the functional enrichment analysis of the DE snoRNAs correlated genes were significantly enriched in 93 KEGG pathways (FDR < 0.05; Supplemental Table S6C) and 1,000 biological process GO-BP terms (FDR < 0.05; Supplemental Table S6D). The top 20 significant KEGG pathways are involved in disease or immune functions including NF-κB signaling pathway, Osteoclast differentiation, TNF signaling pathway, COVID-19, and Chemokine signaling pathway, among others (Fig. 3C) while the top 20 significant GO-BP terms also involved in immune responses included regulation of defense response, response to cytokine, and regulation of immune response, etc. (Fig. 3D).

Interestingly, among all the enriched terms, some disease and immune pathways (e.g., Notch signaling, Fc epsilon RI signaling, Leukocyte transendothelial migration, Neutrophil extracellular trap formation, and T cell receptor signaling) and GO-BP terms (such as cellular response to IL-6, positive regulation of neutrophil extravasation, antigen processing and presentation of peptide antigen via MHC class I, positive regulation of NIK/NF-κB signaling, T-helper 17 type immune response, etc.) were only found in *S. aureus-*positive group (Supplemental Table S6E, S5F) or *S. chromogenes*-positive group (KEGG pathways: IL-17 signaling, p53 signaling, Cell adhesion molecule and Platinum drug resistance etc. and GO-BP terms: negative regulation of cytokine production, negative regulation of interleukin-17 production, negative regulation of I-NF-κB kinase/NF-κB signaling, cellular response to interleukin-4, macrophage differentiation, regulation of Interleukin-12 production, etc. (Supplemental Table S6E, S5F)). Meanwhile, inflammatory and immune pathways common to the *S. chromogenes-*positive and *S. aureus-*positive groups included NOD-like receptor signaling, NF-κB, TNF signaling, chemokine signaling, JAK-STAT signaling, and cancer pathways, while the common GO-BP terms with immune related functions included immune system processes, defense processes, and regulation of cytokine production etc. (Supplemental Table S6E, S5 F).

### Construction of snoRNA-mRNA correlation networks and functional annotation

**Fig. 4:**
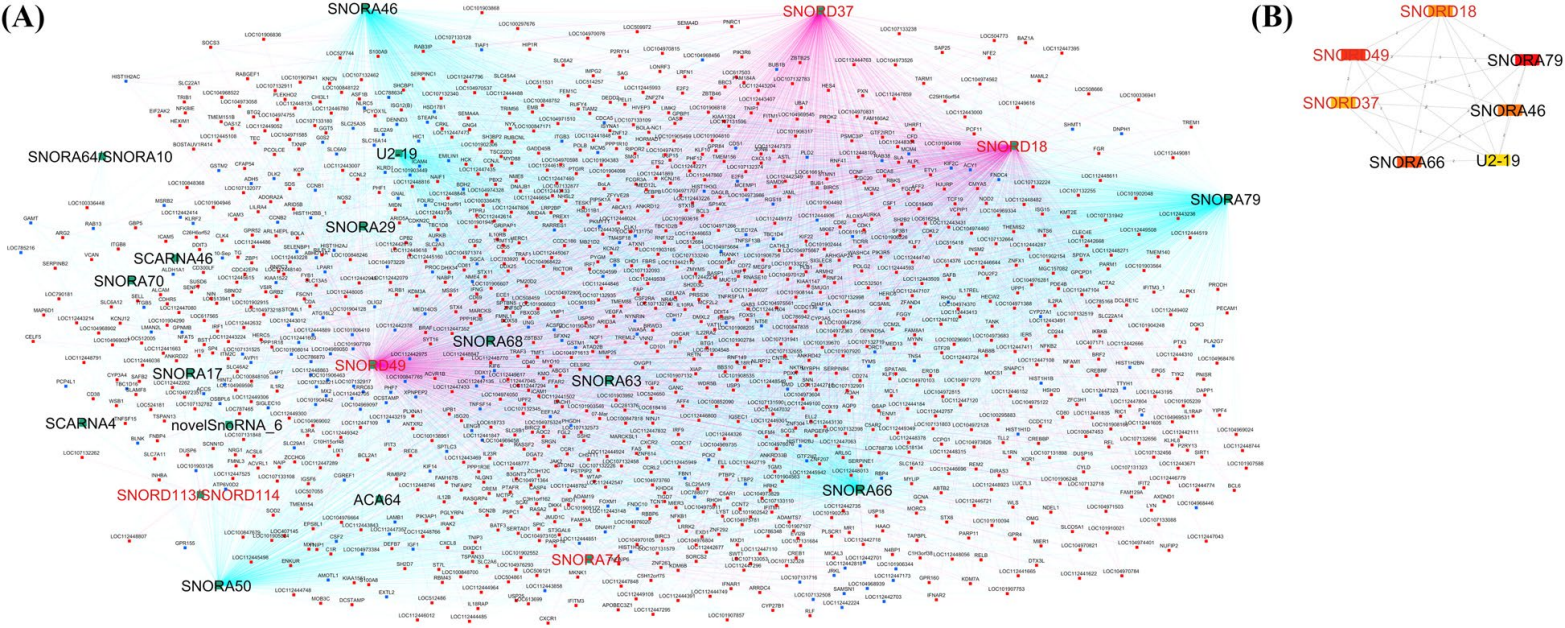
snoRNA-mRNA network for *S. chromogenes-*positive group vs. control group constructed with Cytoscape software. **A** The differentially expressed (DE) snoRNAs connected with their corresponding correlated genes. The downregulated snoRNAs are in red, and the upregulated snoRNAs are in black. The edge colors denote the type of correlation between snoRNAs and mRNAs. The pink edges represent negative correlations between the DE snoRNAs and their correlated genes while the turquoise edges represent positive correlation between the DE snoRNAs and their correlated genes. The green nodes represent the DE snoRNAs. The red nodes represent upregulated mRNAs and the blue nodes represents downregulated mRNAs. **B** Top 7 hub genes in the correlation network identified by CytoHubba (a Cytoscape plugin). Genes with the highest number of connections in the network are considered hub genes. The image shows the degree of importance of the hub snoRNAs through a color scale of rectangular shape ranging from red to yellow. novelSnoRNA_6 is also denoted novelsnoRNA_2_26696606

**Fig. 5:**
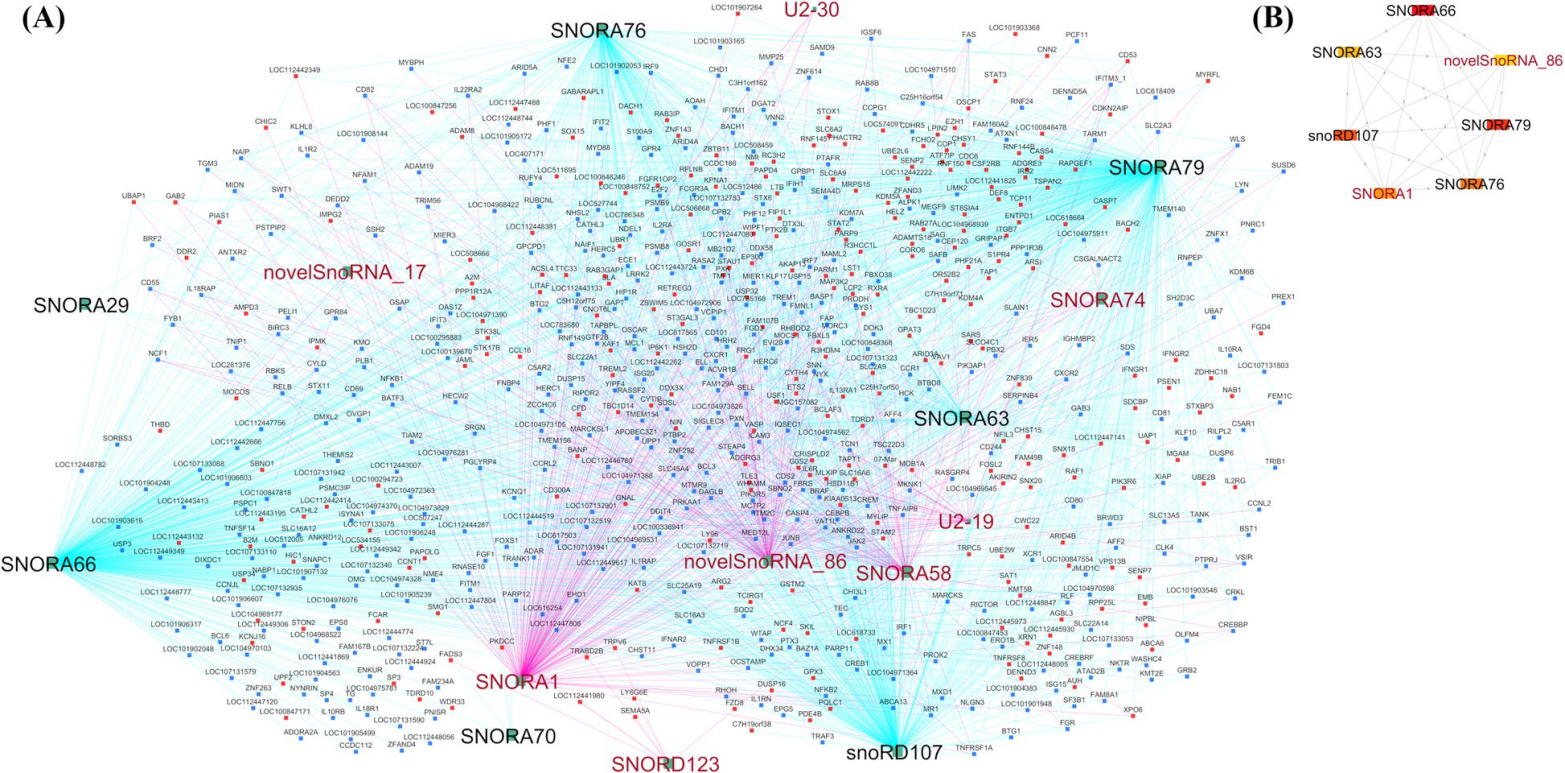
snoRNA-mRNA network for *S. aureus*-positive group vs. control group constructed with Cytoscape software. **A** The differentially expressed (DE) snoRNAs connected with their corresponding correlated genes. The downregulated snoRNAs are in red, and the upregulated snoRNAs are in black. The edge colors denote the type of correlation between snoRNAs and mRNAs. The pink edges represent negative correlations between the DE snoRNAs and their correlated genes while the turquoise edges represent positive correlations between the DE snoRNAs and their correlated genes. The green nodes represent the DE snoRNAs. The red nodes represent upregulated mRNAs and the blue nodes represent downregulated mRNAs. **B** Top 7 hub genes in the correlation network identified by CytoHubba (a Cytoscape plugin). Genes with the highest number of connections in the network are considered hub genes. The image shows the degree of importance of the hub snoRNAs through a color scale of rectangular shape ranging from red to yellow. NovelSonRNA_17 is also denoted novelsnoRNA_4_112140344 and novelsonRNA_86 is also denoted novelsnoRNA_26_14905

To identify snoRNA and mRNA co-expression patterns, snoRNA-mRNA correlation networks were constructed with Cytoscape software. In the *S. aureus-*positive group, 15 DE snoRNAs with correlations to two or more genes resulted in 15 networks (Fig. 4A). From the correlated networks, Cytohubba was further used to identify 7 top hub snoRNAs (snoRNAs correlated with ≥ 100 genes in the network) including upregulated SNORA66 (459 genes), SNORA79 (449 genes), SNORA76 (210 genes), SNORD107 (320 genes), SNORA63 (135 genes) and downregulated novelsnoRNA_26_14905 (117 genes) and SNORA1 (200 genes) (Fig. 4B). Similarly, 18 DE snoRNAs in the *S. chromogenes*-positive group meeting the same criteria resulted to 18 networks (Fig. 5A). From the correlated networks, Cytohubba was further used to identify 7 top hub snoRNAs (snoRNAs correlated with ≥ 30 genes) including upregulated SNORA79 (860 genes), SNORA46 (651 genes), U2-19 (56 genes), SNORA66 (798 genes) and downregulated SNORD37 (37 genes), SNORD49 (31 genes) and SNORD18 (81 genes) (Fig. 5B).

The functional enrichment analysis of the genes correlated with the 7 hub snoRNAs in the *S. aureus*-positive group showed that the GO-BP terms were involved in immune and inflammatory responses, including regulation of defense response, leukocyte differentiation, response to cytokine, regulation of immune response, among others (Supplemental Table S6G). Similarly, the KEGG pathways for this group were also enriched in immune activation and inflammation processes, with key pathways such as Viral protein interaction with cytokine and cytokine receptors, Osteoclast differentiation, NF-κB signaling pathway, JAK-STAT signaling pathway, among others. (Supplemental Table S6H). The top 7 hub SNORNAs correlated genes in the *S. chromogenes*-positive group revealed their GO-BP terms with primarily immune-related functions, such as regulation of defense response, response to cytokine, regulation of response to external stimulus, cellular response to cytokine stimulus, regulation of immune response, and defense response etc. (Supplemental Table S6I). In line with the GO-BP terms, the KEGG pathways for the *S. chromogenes-*positive group involved in immune signaling and inflammatory responses, included Osteoclast differentiation, NF-κB signaling pathway, Viral protein interaction with cytokine and cytokine receptors, TNF signaling pathway, JAK-STAT signaling pathway, and Pathways in cancer etc. (Supplemental Table S6J). Notably, this reveals that the hub SNORNAs in *S. aureus-*positive and *S. chromogenes-*positive groups are potential regulators of inflammatory genes in bovine mastitis.

**Fig. 6:**
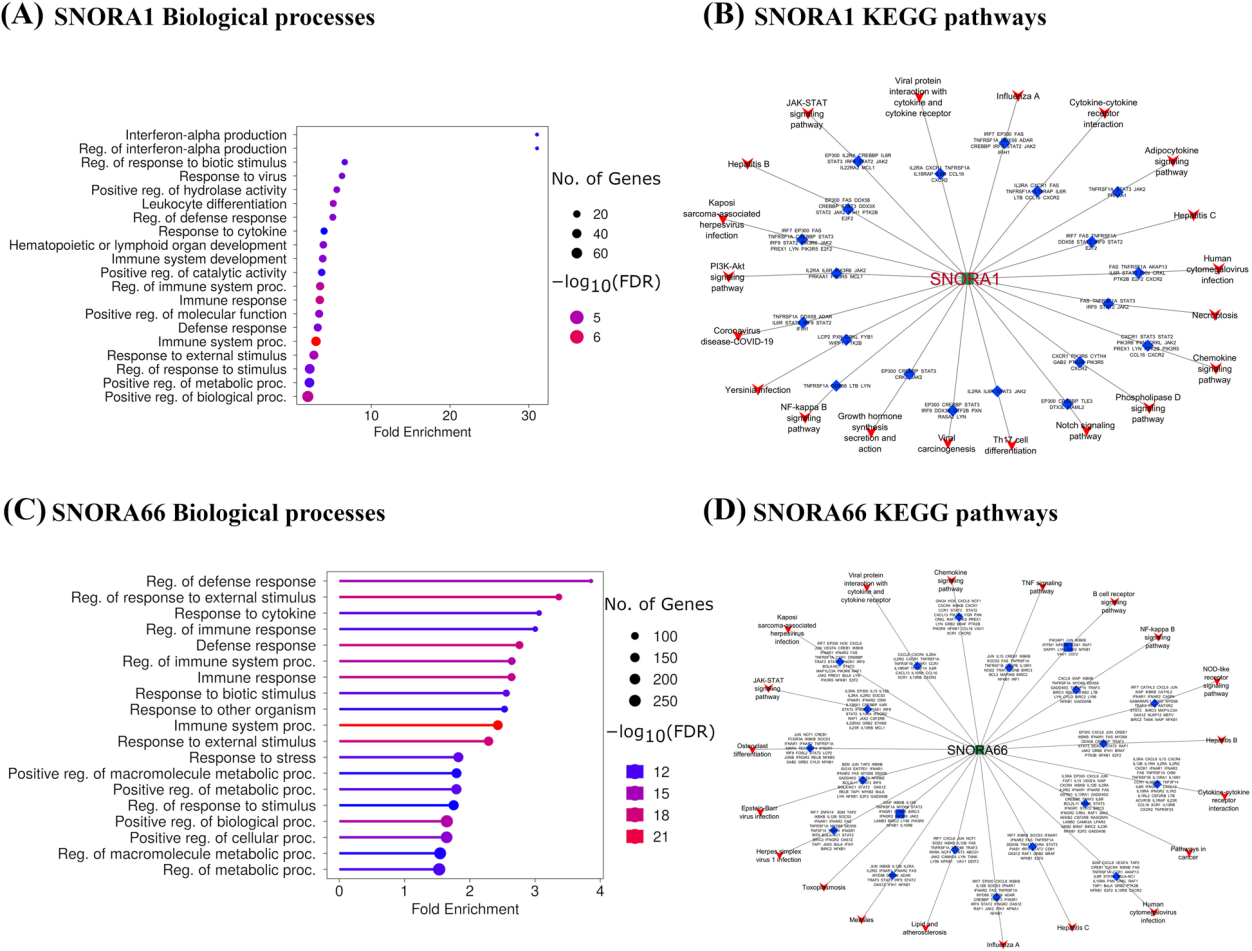
Downregulated SNORA1 and Upregulated SNORA66-correlated mRNA enriched Gene Ontology (GO) biological processes terms for *S. aureus*-positive group. **A** The 20 GO biological processes with the largest gene ratios are plotted in order of gene ratio for SNORA1. The size of the dots represents the number of genes in the significant DE gene list associated with the GO term and the color of the dots represent the *P*-adjusted values (FDR < 0.05). **B** Cytoscape diagram showing the number of genes involved in the top 20 most significant KEGG pathways for SNORA1. The green rectangle shape represents the snoRNA, the blue diamond shape represents the mRNAs, and the red funnel shapes represent the significant pathways. **C** The 20 GO biological processes with the largest gene ratios were plotted in order of gene ratio for SNORA66. The size of the dots represent the number of genes in the significant DE gene list associated with the GO term and the color of the dots represent the *P*-adjusted values (FDR < 0.05). **D** Cytoscape diagram shows the number of genes involved in the top 20 most significant KEGG pathways for SNORA66. The green rectangle shape represents the snoRNA, the blue diamond shapes represent the mRNAs and the red funnel shapes represent the significant pathways

To further understand the roles of the hub snoRNAs, one downregulated snoRNA and one upregulated snoRNA with the highest number of correlated genes in the *S. aureus-*positive and *S. chromogenes-*positive snoRNA-mRNA networks were selected. In the *S. aureus-*positive group, SNORA1 (downregulated) showed correlations with 917 genes (Supplemental Fig. S2A), while SNORA66 (upregulated) was correlated with 1,174 genes (Supplemental Fig. S2B). The top 20 GO-BP terms enriched by genes co-expressed with downregulated SNORA1 were predominantly associated with immune functions, such as leukocyte differentiation, regulation of defense response, response to cytokine, immune system development among others. (Fig. 6A; Supplemental Table S6K). Likewise, over 95% of the top 20 KEGG pathways enriched for these co-expressed genes were involved in immune regulation and disease-related processes including JAK-STAT signaling pathway, P13-AKT signaling pathway, Notch signaling pathway, Chemokine signaling pathway, Th17 cell differentiation, amongst others (Fig. 6B; Supplemental Table S6L). For upregulated SNORA66, 75% of the top 20 GO-BP terms were related to immune functions (e.g., regulation of defense response, regulation of response to external stimulus, response to cytokine, and regulation of immune response (Fig. 6C; Supplemental Table S6M) while the remaining 25% were associated with metabolic and cellular processes (e.g., positive regulation of metabolic process, positive regulation of cellular processes, and regulation of macromolecule metabolic processes). Similarly, the pathways enriched for SNORA66 co-expressed genes, such as Chemokine signaling pathway, Osteoclast differentiation, NF*-κ*B signaling pathway, JAK-STAT signaling pathway, B cell receptor signaling pathway etc. (Fig. 6D; Supplemental Table S6N) were predominantly linked to immune and inflammatory processes. These findings indicate that SNORA1 and SNORA66 may play significant roles in modulating immune responses to infection, highlighting their potential involvement in *S. aureus* subclinical mastitis.

**Fig. 7:**
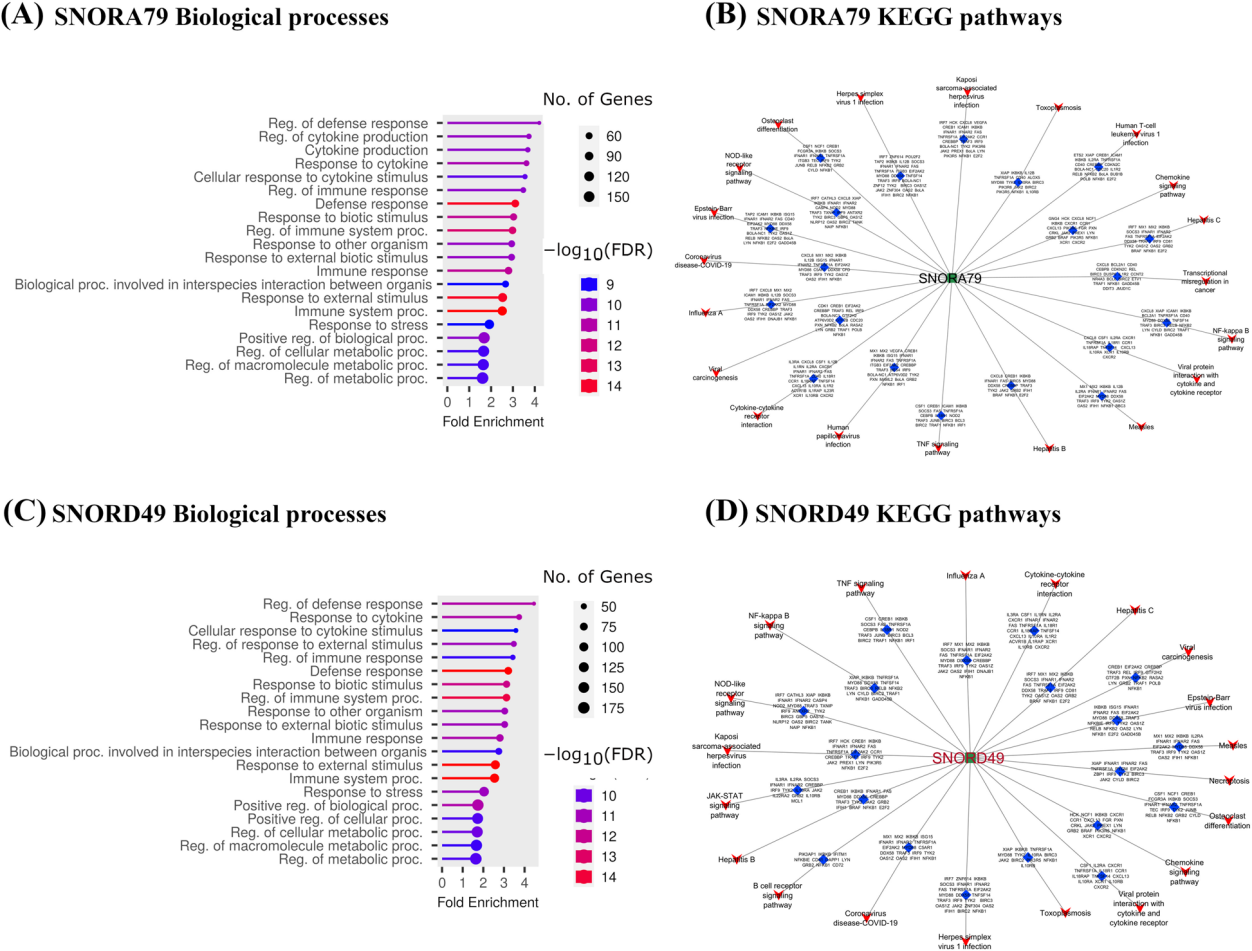
Upregulated SNORA79 and Downregulated SNORD49 correlated mRNAs enriched Gene Ontology (GO) biological processes terms for *S. chromogenes*-positive group. **A** The 20 GO biological processes with the largest gene ratios are plotted in order of gene ratio for SNORA79. The size of the dots represent the number of genes in the significant DE gene list associated with the GO term and the color of the dots represent the *P*-adjusted values (FDR < 0.05). **B** Cytoscape diagram shows the number of genes involved in the top 20 most significant KEGG pathways for SNORA79. The green rectangle shape represents the snoRNA, the blue diamond shapes represent the mRNAs, and the red funnel shapes represent the significant pathways. **C** The 20 GO biological processes with the largest gene ratios were plotted in order of gene ratio for SNORD49. The size of the dots represent the number of genes in the significant DE gene list associated with the GO term and the color of the dots represent the *P*-adjusted values (FDR < 0.05). **D** Cytoscape diagram shows the number of genes involved in the top 20 most significant KEGG pathways for SNORD49. The green rectangle shape represents the snoRNA, the blue diamond shapes represent the mRNAs and the red funnel shapes represent the significant pathways

In the *S. chromogenes*-positive group, one downregulated snoRNA (SNORD49) and one upregulated snoRNA (SNORA79) with the highest number of co-expressed genes in the network were selected. SNORD49 correlated with 1,115 genes (Supplemental Fig. S3A) and SNORA79 correlated with 1,112 genes (Supplemental Fig. S3B). The top 20 enriched GO-BP terms and KEGG pathways for both SNORA79 (Fig. 7A, Supplemental Table S6O; Fig. 7B, Supplemental Table S6P) and SNORD49 (Fig. 7C, Supplemental Table S6Q; Fig. 7D, Supplemental Table S6R) were similar and predominantly related to immune responses including the regulation of defense response, cytokine production, immune response, cellular metabolic processes, among others for the GO-BP terms and NF-κB signaling pathway, TNF signaling pathway and Cytokine-cytokine receptor interaction, etc. for the KEGG pathways. These results suggest that the coordinated regulation of SNORD49 and SNORA79 contributes to a balanced immune response, allowing *S. chromogenes* to coexist with its host without eliciting excessive immune activation. This balance may underlie the pathogen’s milder clinical presentation and greater susceptibility to treatment compared to more virulent pathogens like *S. aureus*.

### Validation of DE snoRNAs using real time quantitative PCR

**Fig. 8:**
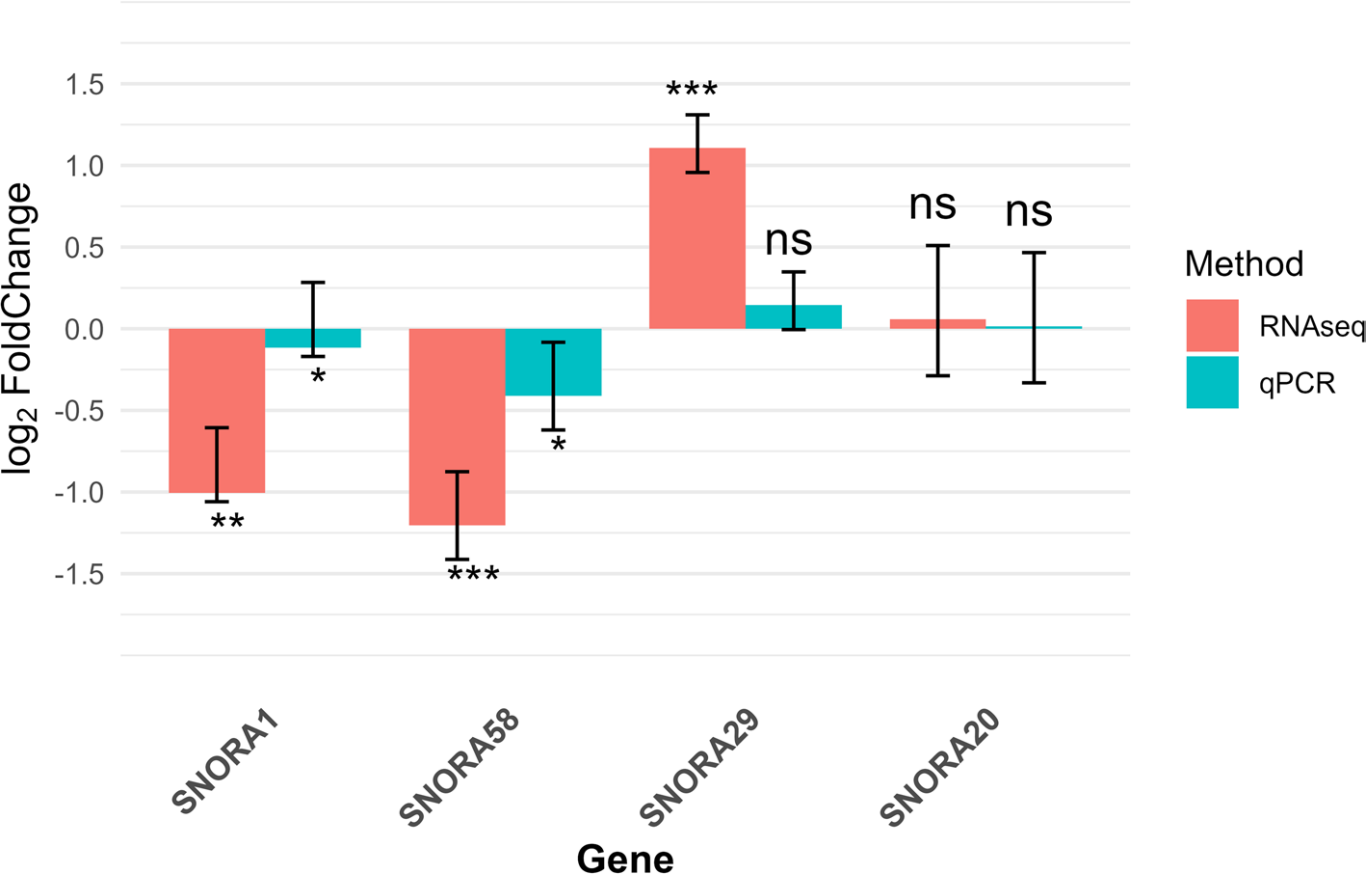
Real-time qPCR expression results compared with snoRNA differential expression results. ^*^P < 0.05, ^**^P < 0.01, ^***^P < 0.001, ^ns^ Non-significant

The real time qPCR expression results of one up-regulated DE snoRNA, two down-regulated DE snoRNAs and one non-DE snoRNA randomly selected to validate the RNA-Seq results are shown in Fig. 8. The qPCR relative expression results for the tested snoRNAs were similar to the RNA-Seq expression data for the two downregulated snoRNAs and the one non-DE snoRNA. However, the expression of the upregulated snoRNA was not significant as compared to the RNA-Seq results (Fig. 8).

## Discussion

Through transcriptome analysis of milk somatic cells from cows with naturally occurring subclinical mastitis due to *S. aureus* and *S. chromogenes*, 255 snoRNAs (233 known and 22 novel; Supplemental Table S2) were identified. Out of the identified snoRNAs, 21 were DE (11 upregulated and 10 downregulated) in *S. aureus*-positive group (Fig. 2A) whereas 20 were DE in *S. chromogenes*-positive group (Fig. 2B). SnoRNAs are crucial for rRNA modification and essential for ribosome function, as highlighted in previous studies [13, 14]. In our study, 2′-O-methylation modification sites on 18S rRNA and 28S rRNA were observed for majority of the DE snoRNAs in *S. aureus*-positive and *S. chromogenes*-positive groups (Tables 1 and 2). Interestingly, there were also some snoRNAs having modification sites common between the two groups on both 18S and 28S rRNA pseudouridylation sites, such as SNORA29 (18S-1323, 28S-4262); SNORA66 (18S-1323, 28S-4232); SNORA74 (18S-596, 28S-263) etc. (Tables 1 and 2). Moreso, some snoRNAs were found to target the same modification sites on 18S rRNA, such as SNORA70, SNORA66 and SNORA76 (18S-1323) for *S. aureus*-positive group (Table 1). These findings are consistent with human studies, where snoRNAs like SNORA63, SNORA70, and SNORA1 have been shown to guide chemical modifications of rRNA processing [34, 35] and control translation processes such as initiation, elongation, and termination [19, 36, 37], suggesting their vital role during subclinical mastitis.

The observed DE snoRNAs in the *S. aureus-*positive group (including U3, U5 snRNA, SNORA74, SNORA66, SNORD123, SNORA1 and novelsnoRNA_26_14905 (log_2_FC > 1 or log_2_FC < −1) among others), and in the *S. chromogenes*-positive group (including SNORD113/SNORD114, SNORA66, SNORD49 and SNORA46, etc. (log_2_FC > 1 or log_2_FC < −1)) are consistent with findings from previous research [38–42]. For instance, Li and colleagues found upregulated expression of SNORA66 in human patients with diffuse large B-cell lymphomas [42]. Ferreira and colleagues reported that SNORD123 was silenced in various cancers due to hypermethylation of their host gene-associated CpG islands, indicating a novel subclass of non-coding RNAs targeted in tumorigenesis [40]. In another study, snoRNA expression data from eight major cancers revealed that SNORD123 contributed to high classification accuracy, with the support vector machines classifier showing a Matthew's correlation coefficient of 0.881 for identifying cancer samples suggesting its importance in understanding tumor biology and developing targeted therapies [41].​ In a recent study, overexpression of SNORA76 was identified as a potential biomarker for early detection of clear cell renal cell carcinoma from urine-derived extracellular vesicles [38]. Furthermore, SNORA76 was found to be upregulated in gallbladder cancer compared to adjacent non-tumor tissues [39] and significantly downregulated in metastatic vs. non-metastatic prostate cancer xenograft models [43]; SNORD37 was found to be downregulated in non-small cell lung cancer [44] while SNORA64 was identified as significantly upregulated in pancreatic cancer patients [45] and acute myeloid leukemia patients [46]. Overall, these findings suggest the involvement of these snoRNAs in disease processes and consequently in *S. aureus* and *S. chromogenes* subclinical mastitis. Furthermore, 10 DE snoRNAs identified in this study were found to be common between the two pathogens, including SNORA66, SNORA70, SNORA29, and ACA64, suggesting involvement in common biological processes. Meanwhile some snoRNAs displayed pathogen-specific expression patterns, such as SNORD123, SNORA58, and SNORA76 in *S. aureus-*positive group and SNORA50, SNORA68, and SNORD49 in *S. chromogenes-*positive group (Fig. 2C), suggesting differential regulatory mechanisms between *S. aureus* and *S. chromogenes*-induced mastitis.

To further explore the roles of the DE snoRNAs, the negative and positive correlated DE mRNAs from each group (correlation coefficient > 0.9; Supplemental Table S4A and S4B) were subjected to functional enrichment. Both positive and negative correlations were observed, and which reflects the dual roles of snoRNAs in gene regulation. These roles include their positive involvement in rRNA processing and biogenesis as well as their non-canonical regulatory functions, such as repressing the expression of specific mRNAs or reflecting a feedback-loop [16]. In both *S. aureus-*positive and *S. chromogenes-*positive cows, the DE snoRNAs were correlated with several immune and inflammatory genes (e.g., *IL1R*,* IL18R1*,* STAT3*,* NFKB2*,* MYD88*,* VEGFA* and *CD40*), all of which have been reported in several studies to be associated to mastitis [47–53]. As evident in our study, the enrichment analysis of the correlated genes showed that the top 20 KEGG pathways and GO-BP were enriched in immune and inflammatory functions including NF-κB signaling, TNF signaling, immune system processes and defense responses, among others (Fig. 3A and B). These pathways and biological processes terms have been associated to the immune response to mastitis pathogens through analysis of different biological molecules like mRNA, miRNA and lncRNA [7, 30, 54], thus supporting the involvement of snoRNAs in the host immune response to *S. aureus* and *S. chromogenes* induced mastitis.

For the *S. aureus*-positive group, some enriched pathways and biological processes terms (Supplemental Table S6E, S5F) are indicative of *S. aureus*’s reliance on complex regulatory pathways (e.g., Leukocyte transendothelial migration, and Neutrophil extracellular trap (NET) formation) to enhance immune activation and establish infection. Generally, neutrophils which make up to 75% of the leukocytes in milk from cows with mastitis, exhibit anti-inflammatory activity through three main mechanisms: phagocytosis, degranulation, and the release of NETs. NETs are large, extracellular, web-like structures made up of cytosolic and granule proteins organized on a scaffold of decondensed chromatin and their dysregulation can lead to tissue damage, contributing to the development of immune-related diseases [55].

For the *S. chromogenes*-positive group, the correlated genes (including *IFNG, IL12B*, etc.) were involved in immune and cellular pathways such as IL-17 signaling, p53 signaling, Cell adhesion molecule pathways etc., while some GO-BP terms were mostly involved in negative regulation of the immune processes such as negative regulation of cytokine production, negative regulation of IL-17 production, cellular response to IL-4, and macrophage differentiation among others (Supplemental Table S6E, S5F). Among the correlated genes involved in these processes, the *IFNG* gene encodes interferon-gamma (*IFN-γ*), a critical cytokine in the immune system. Interferon-gamma is a small but highly potent molecule widely utilized in clinical treatments due to its role in modulating immune responses and enhancing resistance to infections. During *Staphylococcus* infections, *IFN-γ* is produced by capsular polysaccharide-stimulated T cells, amplifying the harmful effects on resistant *Staphylococcus* strains [56]. Interferon-gamma has been used to manage acute bovine mastitis during the periparturient period. Its application induces functional changes in lymphocytes and phagocytic cells within the mammary gland, leading to decreased mastitis severity [57]. In this study, the negative regulation of the IL-17 pathway suggests that it may be a strategy to keep inflammation under control [58]. It is generally considered that interleukin 17 pathway could activate antimicrobial peptides and neutrophil activating molecules [59]. Also, they could improve host innate and adaptive defense by modulating the gene expression patterns of mammary gland epithelial cells [59, 60]. These results reflect *S. chromogenes*’s lower virulence and the milder immune activation it induces.

Interestingly, we observed that some of the significant pathways and GO terms were found in the *S. aureus*-positive group only or the *S. chromogenes* positive group only. This observation is not surprising as it is a well-established fact that most of the mastitis infections due to *S. aureus* develop to a more severe and prolonged infection while those due to *S. chromogenes* are milder [61–64] and could explain the differences in the observed pathways. Moreover, Wang and colleagues reported mostly positive and higher gene set scores for immune-related pathways and GO terms in *S. aureus-*positive cows supporting a more aggressive profile which also aligns with *S. aureus* higher virulence and robust immune evasion strategies that allow it to persist in the mammary gland and cause severe infections [30]. Meanwhile, mostly negative and lower gene set scores for the corresponding terms were reported in relation to *S. chromogenes-*positive cows supporting a less aggressive profile [30]. Likewise, several KEGG pathways and biological processes terms related to immune and inflammatory functions identified (e.g., NOD-like receptor signaling, NF-κB signaling, TNF signaling, Chemokine signaling, JAK-STAT signaling, defense responses, and regulation of cytokine production; Supplemental Table S6E, S5F) were associated to both *S. aureus* and *S. chromogens* suggesting that both pathogens belonging to the staphylococci genus use some common mechanisms to manipulate the host immune response to establish subclinical mastitis and persist within the host.

To gain further insights into the functions of DE snoRNAs, a cytoscape plugin was used to construct snoRNA-mRNA co-expressed networks for the two pathogens (Supplemental Table S4). Majority of the correlated genes have immune functions suggesting that snoRNAs may play a role in their regulation. In particular, further identification of hub snoRNAs (Figs. 4A, B and 5A, B) support a role of the DE snoRNAs in regulating many genes in the networks. Furthermore, the functional enrichment analysis of the correlated genes of the 7 hub snoRNAs in *S. chromogenes-*positive group (SNORD18, SNORA79, SNORA46, U2-19, SNORA66, SNORD37, SNORD49) revealed involvement in immune pathways (NF-κB, JAK-STAT, IL-17, TNF-signaling pathways; Supplemental Table S6G) and mostly negative immune and cellular biological processes (e.g., negative regulation of immune response, negative regulation of leucocyte activation, negative regulation of inflammatory responses; Supplemental Table S6H). This further buttresses a mild host immune response to the infection by *S. chromogenes*. Piccart et al. found that dairy heifers inoculated with *S. chromogenes* strains in the mammary gland evoked a mild local host response [65]. Similarly, Simojoki and colleague revealed that *S. chromogenes* infected primiparous cows had minor tissue damage and the infection was eradicated in few days [63]. However, since *S. chromogenes* is the most NAS pathogen frequently isolated from milk and skin, the prevalence and re-occurrence of the infection may be more problematic [63], requiring further investigation.

In contrast to *S. chromogenes-*positive group, the *S. aureus-*positive group correlated genes of the 7 hub snoRNAs (SNORA66, novelsnoRNA_26_14905, SNORD107, SNORA1, SNORA63, SNORA79 and SNORA76) were mostly involved in positive regulation of immune and inflammatory responses including positive regulation of immune system process, positive regulation of cytokine production, positive regulation of leucocyte differentiation, regulation of autophagy, NF-κB, JAK-STAT and TNF-signaling pathways, among others (Supplemental Table S6I, S5J). The positive regulation of the immune responses could explain *S. aureus* pathogenicity to evade both innate and adaptive immune responses thereby causing localized or systemic infections [66]. *S. aureus* has the ability to internalize, multiply and persist in bovine mammary gland thereby making *S. aureus* mastitis infection difficult to eradicate [67]. Among the mechanisms underlying the intracellular survival of *S. aureus* is its ability to induce autophagy formation. In this study, *STAT3* reported to regulate autophagy [68] was enriched in autophagy GO-BP term. Following infection with *S. aureus*, the activation of *STAT3* occurs later than other signaling pathways, such as *ERK* and *mTOR*, leading to the delayed suppression of *TFEB*, a key regulator of lysosomal biogenesis and function. This delayed *STAT3* activation results in a weakened lysosomal function in macrophages, impairing their ability to degrade and clear the bacteria. By inhibiting *TFEB* activity, *STAT3* facilitates the survival of *S. aureus* within macrophages, allowing the bacteria to evade the host’s immune response [68]. This study highlights the strategic manipulation of host cell pathways by *S. aureus* to prevent bacterial clearance and promote persistent infection. These findings suggest the potential roles of the 7 hub snoRNAs in regulating inflammatory responses during mastitis. However, while our correlation and enrichment analyses suggest that certain snoRNAs may be involved in immune-related pathways during subclinical mastitis, the regulatory direction and mechanism remain to be validated. Most snoRNAs are known to function in trans [13], but their emerging roles in mRNA regulation remain to be investigated. Similarly, we observed strong correlations between some snoRNAs and immune-related genes. However, future studies involving snoRNA perturbation (e.g., knockdown or overexpression) are essential to confirm the regulatory roles of these snoRNAs, and establish causality.

The correlated genes (e.g., *JAK2*,* SOCS3*,* PIK3AP1*,* PTK2B*; Supplemental Fig. S2–S3) of the 7 hub SNORNAs for *S. aureus*-positive group and *S. chromogenes-*positive group were also enriched in metabolic processes and pathways related to milk synthesis, such as response to lipid, prostaglandin metabolic process, prolactin signaling pathway and JAK-STAT signaling pathway, among others (Supplemental Table S6K–R). The JAK-STAT pathway plays central roles in regulating cytokine signaling in the mammary gland with strong association with mammary gland development and milk production [69]. This pathway is crucial for blood cell differentiation and the regulation of casein gene expression during lactation [70, 71]. For instance, JAK-STAT-associated proteins, regulated by prolactin receptor, help maintain a balance between growth hormone function and milk protein synthesis [72]. The interplay between lipid-related processes and immune responses during subclinical mastitis caused by *S. aureus* or *S. chromogenes* could lead to reduced milk fat content, altered milk composition, and a shift in energy allocation towards supporting immune functions [30]. These findings highlight potential regulatory mechanisms underlying the compromised milk production observed in cows with subclinical mastitis, emphasizing the complex molecular interactions driving these changes.

Furthermore, the roles of the top downregulated or upregulated hub snoRNAs in each of the pathogen groups were investigated through functional analyses. In the *S. aureus-*positive group, the correlated genes of the 2 hub SNORNAs (upregulated SNORA66 and downregulated SNORA1; Fig. 6) including inflammatory cytokines (e.g., *CXCL8*,* IL1R*,* IL-10R*,* IFNGR1*; Supplemental Fig. S2A) and chemokine (e.g., *STAT3*,* IL6R*,* IL2*,* JAK2*; Supplemental Fig. S2B) genes show involvement in immune responses and pathways like JAK-STAT signaling pathway, P13K-AKT signaling pathway, Th17 differentiation, Leukocyte differentiation, Interferon alpha production, Positive regulation of interleukin 6 production and neutrophil chemotaxis and extravasation etc. (Supplemental Table S6K–N). The genes correlated with downregulated SNORA1 (e.g., *JAK2* and *IL6R*) have been shown to stimulate inflammation during subclinical mastitis as discussed in several reviews [54, 60, 73, 74]. For instance, IL-6 receptor (*IL6R*) is a receptor that transmits the effects of *IL-6,* a cytokine involved in promoting inflammation [60]. Bochniarz et al. found upregulated expression of *IL-6* in *Streptococcus* spp. infected cows, suggesting that it is an important cytokine that could be used for early detection of subclinical mastitis [75]. *JAK-2* is one of the key members in JAK-STAT signaling pathway and the activation of this gene is critical in bovine mastitis resistance. In contrast, the correlated chemokines and cytokines genes (e.g., *CXCL8* and *IL-10R*) of upregulated SNORA66 are mostly involved in the protection against pathogens infecting bovine mammary glands through recruiting leukocytes from blood into the mammary tissue [76–79]. For instance, *IL-10* could exhibit immune-stimulatory properties, aiding in the clearance of infectious and noninfectious particles while minimizing inflammation [80, 81]. *CXCL8* (also known as interleukin-8) plays a pivotal role in the immune response by directing the migration and activation of neutrophils. It is the most potent neutrophil-attracting chemokine to infections and tissue injuries. In the *S. chromogenes* positive group, the correlated genes of the two hub SNORNAs (downregulated SNORD49 and upregulated SNORA79; Fig. 7) suggest they may mediate the upregulation of various pro-inflammatory genes (e.g., *CXCL8*,* IKBKB*,* TRAF3*,* NFKB1*; Supplemental Fig. S3) which are involved in bridging innate and adaptive immunity by regulating inflammation locally and systematically via the recruitment of neutrophils and macrophages to the site of infection [73] via the IL-17 signaling pathway and p53 signaling pathway among others (Supplemental Table 6O–R). These genes have been reported to increase in response to bovine mastitis pathogens [30, 82–84].

This study provides valuable data for understanding the snoRNAs regulatory mechanisms behind subclinical mastitis caused by *S. aureus* and *S. chromogenes.* However, extrapolation of our results needs to be careful, due to the relatively small sample sizes. While we used stringent thresholds to identify robustly expressed snoRNAs (read counts ≥ 10 in all samples and log_2_FC > 1) and a software (DESeq2 [24]) that is specifically designed to handle small and unbalanced RNA-seq datasets, further validation of the functional roles of the identified snoRNAs using larger and more balanced datasets is necessary to confirm our findings. Moreover, experimental validation of snoRNA-mRNA interactions and predicted rRNA modification sites (e.g., via mass spectrometry) is essential and represents an important direction for future functional characterization.

## Conclusion

This study explored the roles of dysregulated snoRNAs in subclinical mastitis caused by *S. aureus* and *S. chromogenes*. In this study, 2′-O-methylation and pseudouridylation binding sites on 18S rRNA and 28S rRNA were observed for majority of the DE snoRNAs in *S. aureus*-positive and *S. chromogenes*-positive groups, suggesting snoRNA potential roles in rRNA biogenesis, translation and processing during bovine subclinical mastitis. Likewise, the DE snoRNAs were associated with key immune and inflammatory pathways and processes including NF-κB, JAK-STAT, TNF signaling pathways and leucocyte activation, among others. Moreover, correlated mRNAs in snoRNA-mRNA networks for the two pathogens have mostly immune related functions and correlated genes of the hub snoRNAs identified for *S. aureus*-positive and *S. chromogenes*-positive groups suggest important roles in regulating immune and inflammatory pathways and processes through overrepresentation in IL-17 signaling pathway and p53 signaling pathway, etc., and JAK-STAT signaling pathway, P13K-AKT signaling pathway, Th17 differentiation pathway and Leukocyte differentiation pathway, among others, respectively.

## Supplementary Information

Supplementary Material 1. Table S1 General whole genome snoRNA sequence statistics per sample.

Supplementary Material 2. Table S2 List of identified snoRNAs expressed in samples.

Supplementary Material 3. Table S3A List of differntially expressed snoRNAs in *Staphylococcus aureus* versus control group. Table S3B List of differntially expressed snoRNAs in *Staphylococcus chromogenes* versus control group.

Supplementary Material 4. Table S4A Summary of snoRNA function and their rRNA target modification site in *Stahylococcus aureus* versus control group. Table S4B Summary of snoRNA function and their rRNA target modification site in *Stahylococcus chromogenes* versus control group.

Supplementary Material 5. Table S5A Correlation anaysis of differentially expressed (DE) snoRNAs and DE mRNAs in *Staphylococcus aureus* versus control group. Table S5B Correlation anaysis of differentially expressed (DE) snoRNAs and DE mRNAs in *Staphylococcus chromogenes* versus control group.

Supplementary Material 6. Table S6A Functional enrichment Gene Ontology Biological processes of differentially expressed snoRNA correlated genes of *Staphylococcus aureus* versus control group. Table S6B Functional enrichment Gene Ontology Biological processes of differentially expressed snoRNA correlated genes of *Staphylococcus chromogenes* versus control group. Table S6C Functional enrichment KEGG pathway analysis of differentially expressed snoRNA correlated genes of *Staphylococcus aureus* versus control group. Table S6D Functional enrichment KEGG pathway analysis of differentially expressed snoRNA correlated genes of *Staphylococcus chromogenes* versus control group. Table S6E Functional enrichment KEGG pathway analysis of differentially expressed snoRNA correlated genes specific to *Staphylococcus aureus*, *Staphylococcus chromogenes* and *Staphylococcus aureus* versus *Staphylococcus chromogenes*. Table S6F Functional enrichment Gene Ontology Biological processes terms of differentially expressed snoRNA correlated genes specific to *Staphylococcus aureus*, *Staphylococcus chromogenes* and *Staphylococcus aureus* versus *Staphylococcus chromogenes*. Table S6G Functional enrichment KEGG pathways of 7 hub snoRNAs correlated genes of *Staphylococcus chromogenes* versus control group. Table S6H Functional enrichment Gene Ontology Biological processes of 7 hub snoRNAs correlated genes of *Staphylococcus chromogenes* versus control group. Table S6I Functional enrichment Gene Ontology Biological processes of 7 hub snoRNAs correlated genes of *Staphylococcus aureus* versus control group. Table S6J Functional enrichment KEGG pathways of 7 hub snoRNAs correlated genes of *Staphylococcus aureus* versus control group. Table S6K Functional enrichment Gene Ontology Biological processes of SNORA1 correlated genes of *Staphylococcus aureus* versus control group. Table S6L Functional enrichment KEGG pathway analysis of differentially expressed SNORA1 correlated genes of *Staphylococcus aureus* versus control group. Table S6M Functional enrichment Gene Ontology Biological processes of SNORA66 correlated genes of *Staphylococcus aureus* versus control group. Table S6N Functional enrichment KEGG pathway analysis of differentially expressed SNORA66 correlated genes of *Staphylococcus aureus* versus control group. Table S6O Functional enrichment Gene Ontology Biological processes of SNORA79 correlated genes of *Staphylococcus chromogenes* versus control group. Table S6P Functional enrichment KEGG pathway analysis of differentially expressed SNORA79 correlated genes of *Staphylococcus aureus* versus control group. Table S6Q Functional enrichment Gene Ontology Biological processes of SNORD49 correlated genes of *Staphylococcus chromogenes* versus control group. Table S6R Functional enrichment KEGG pathway analysis of differentially expressed SNORD49 correlated genes of *Staphylococcus aureus* versus control group.

Supplementary Material 7. Table S7 Genes and their primer sequences used for real-time qPCR validation of RNA-sequencing results.

Supplementary Material 8. Fig. S1 Principal component analysis plot showing the sample cluster per group. Fig. S2 Correlation network between hub snoRNAs and target DE genes for *Staphylococcus aureus* versus the control. Fig. S3 Correlation network between hub snoRNAs and target DE genes for *Staphylococcus chromogenes* versus the control.

## Data Availability

The raw read sequences analyzed in this study have been deposited in NCBI Sequence Read Archive (SRA) under the Bio Projects PRJNA878880 and PRJNA967255.

## Abbreviations

AML: Acute myeloid leukemia
APA: Alternative polyadenylation
*CDC2L6*: Cyclin-dependent kinase 6
*CXCL8*: Chemokine (C-X-C motif) ligand 8
*CXCR1*: Chemokine receptor 1
*CXCR2*: Chemokine receptor 2
DE: Differentially expressed
DHI: Dairy herd improvement
DNA: Deoxyribonucleic acid
FDR: False discovery rate
*G3BP1*: Ras‐GTPase‐activating protein (GAP)‐binding protein 1
GFF: General feature format
GO: Gene Ontology
HC: Healthy control
*IFNGR1*: Interferon gamma receptor 1
*IKBKB*: Inhibitor of nuclear factor kappa B kinase subunit beta
IL-17: Interleukin 17
*IL1R*: Interleukin receptor 1
*IL6R*: Interleukin receptor 6
I-NF-κB kinase/NF-κB: I-κB kinase (IKK) and Nuclear Factor kappa B (NF-κB)
*JAK2*: Janus kinase 2
JAK-STAT: Janus kinase/signal transducers and activators of transcription
KEGG: Kyoto Encyclopedia of Genes and Genomes
Log_2_FC: Log_2_ fold change
LncRNAs: Long non coding RNAs
MEGA: Molecular Evolutionary Genetics Analysis
MHC: Major histocompatibility complex
miRNAs: MicroRNAs
mRNAs: Messenger RNAs
MUSCLE: Multiple sequence comparison by log-expectation
NAS: Non-aureus staphylococci
ncRNAs: Non-coding RNAs
NET: Neutrophil extracellular trap formation
NF-κB: Nuclear factor kappa B
ON: Ontario
P13-AKT: Phosphatidylinositol 3-kinase (PI3K) and protein kinase B
PBS: Phosphate buffered saline
PCA: Principal component analysis
PCR: Polymerase chain reaction
*PIK3AP1*: Phosphoinositide-3-kinase adaptor protein 1
*PTK2B*: Protein tyrosine kinase 2 beta
QC: Quebec
RIN: RNA integrity number
RNA: Ribonucleic acid
rRNA: Ribosomal RNA
SCC: Somatic cell count
SCS: Somatic cell score
snoRNA: Small nucleolar RNA
*SOCS3*: Suppressor of cytokine signaling 3
STAR: Spliced transcripts alignment to a reference
*STAT3*: Subunit signal transducers and activators of transcription 3
*TNF*: Tumor necrosis factor
*TNFRSF1A*: Tumor necrosis factor receptor superfamily member 1A
*TRAF1*: Tumor necrosis factor receptor associated factor 1
*TRAF3*: Tumor necrosis factor receptor associated factor 3
*UBAP2L*: Ubiquitin associated protein 2 like
UMI: Unique molecular identifiers
