# Supplementary material for: Small nucleolar RNA dysregulation and potential roles in bovine subclinical mastitis
Source: J Anim Sci Biotechnol. 2025 Sep 12;16:124. doi: 10.1186/s40104-025-01230-9 (PMC12427106; doi:10.1186/s40104-025-01230-9)
Supplement: Supplementary file 8 — Supplementary Material 8. Fig. S1 Principal component analysis plot showing the sample cluster per group. Fig. S2 Correlation network between hub snoRNAs and target DE genes for Staphylococcus aureus versus the control. Fig. S3 Correlation network between hub snoRNAs and target DE genes for Staphylococcus chromogenes versus the control. [file 40104_2025_1230_MOESM8_ESM.pdf]

Supplemental figures

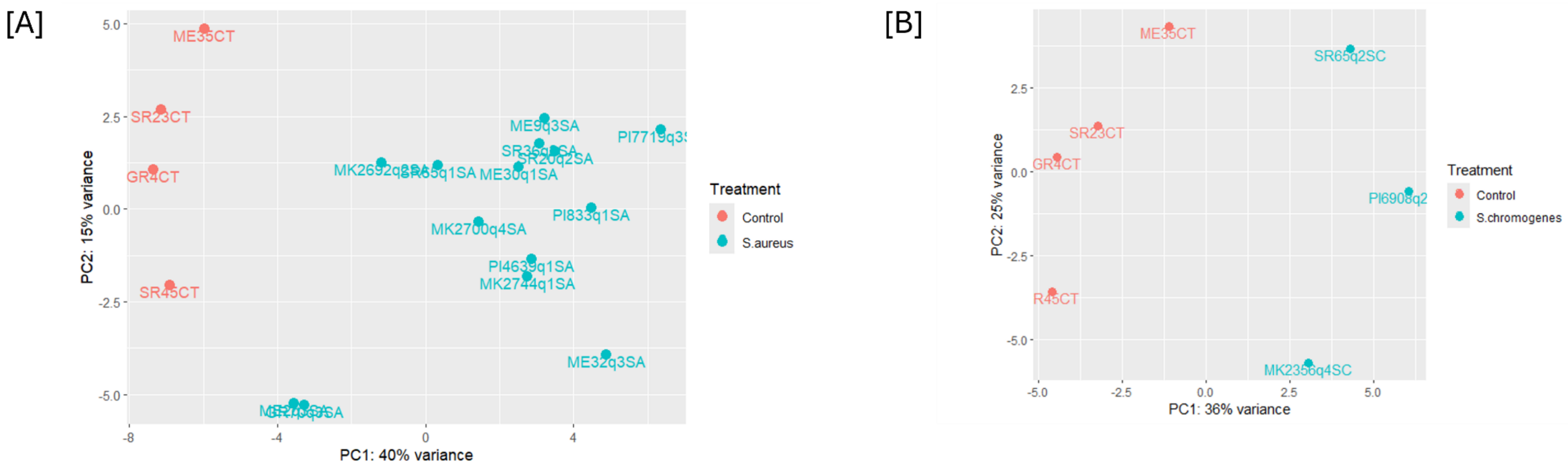

Figure S1. Principal component analysis plot showing the sample cluster per group. (A) PCA plot between *Staphylococcus aureus* versus control group. (B) *Staphylococcus chromogenes* versus the control group. The red color represents the control group and the turquoise color represents the pathogen group.

[2A]

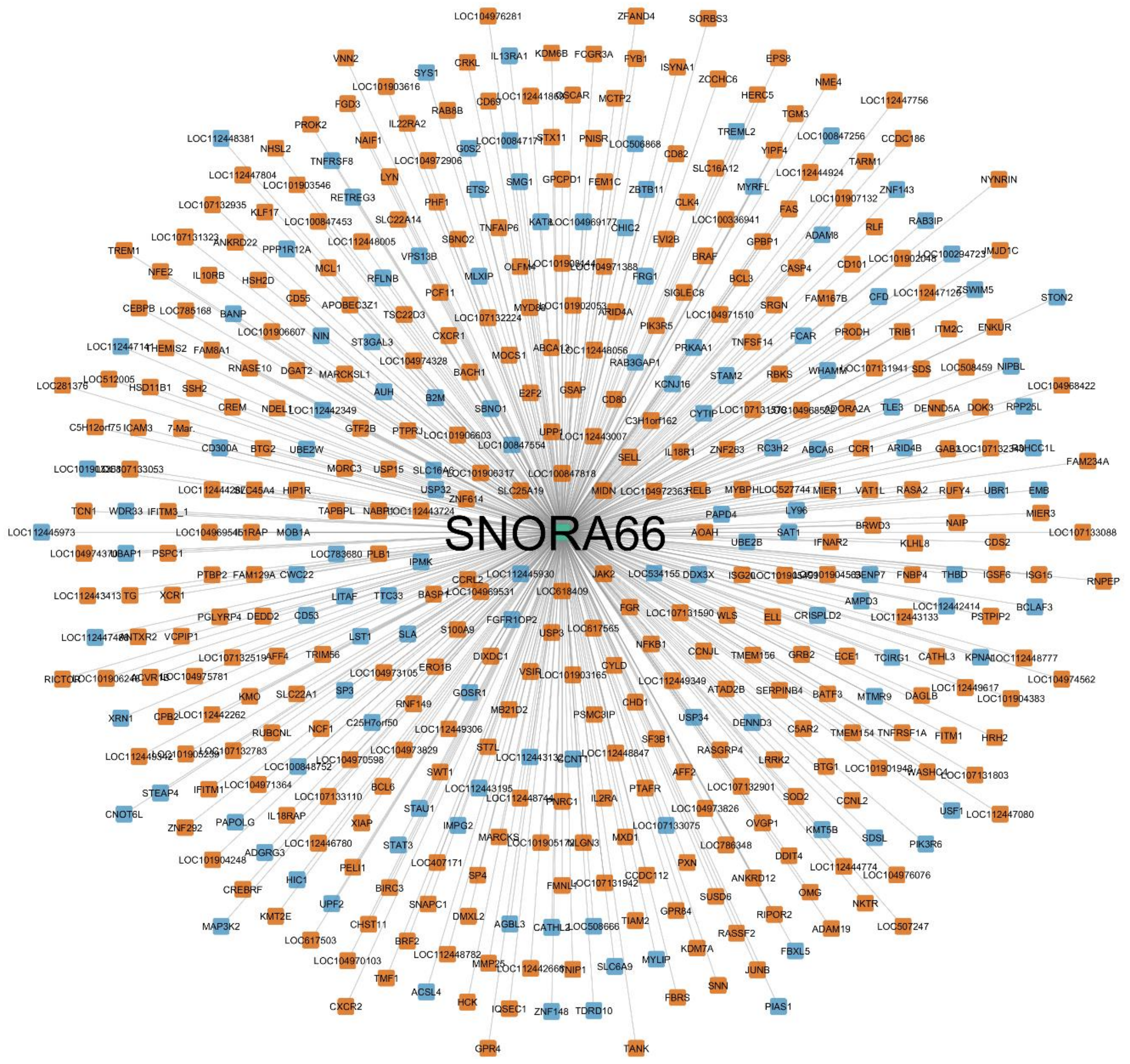
SNORA66
LOC104976281
ZFAND4
SORBS3
VNN2
SYS1
CRKL
IL13RA1
KDM6B
FCGR3A
FYB1
ISYNA1
ZCCHC6
EPS8
NME4
LOC101903616
FGD3
RAB8B
CD69
MCTP2
HERC5
TGM3
LOC112447756
PROK2
IL22RA2
NAIF1
LOC112448381
NHSL2
TNFRSF8
LOC104972906
STX11
PNISR
LOC506868
TREML2
LOC100847256
CCDC186
LYN
CD82
SLC16A12
YIPF4
TARM1
LOC112447804
LOC101903546
ETS2
SMG1
GPCPD1
FEM1C
ZBTB11
MYRFL
LOC112444924
NYNRIN
RETREG3
PHF1
LOC107132935
KLF17
LOC100847453
SLC22A14
TNFAIP6
CHIC2
CLK4
LOC100336941
FAS
LOC107907132
ZNF143
RAB3IP
ANKRD22
PPP1R12A
LOC112448005
SBNO2
EVI2B
GPBP1
ADAM8
RLF
TREM1
NFE2
IL10RB
MCL1
VPS13B
MLXIP
OLFM4
LOC104971388
BRAF
CASP4
CD101
JMJD1C
HSH2D
RFLNB
FRG1
BCL3
PCF11
SIGLEC8
SRGN
FAM167B
CFD
ZSWIM5
STON2
CEBPB
LOC785168
BANP
CD55
APOBEC3Z1
TSC22D3
MYD88
ARID4A
PIK3R5
LOC104971510
TRIB1
ITM2C
ENKUR
LOC107132224
LOC101906607
NIN
ST3GAL3
CXCR1
LOC112448056
PRKAA1
TNFSF14
FCAR
PRODH
THEMIS2
FAM8A1
LOC104974328
MOCS1
ABCA1
RAB3GAP1
STAM2
RBKS
WHAMM
SDS
LOC508459
NIPBL
RNASE10
DGAT2
MARCKSL1
BACH1
LOC104968422
LOC512005
HSD11B1
SSH2
AUH
B2M
SBNO1
E2F2
GSAP
CD80
KCNJ16
CREM
LOC112442349
TLE3
DENND5A
DOK3
RPP25L
ICAM3
GTF2B
PTPRJ
LOC101906603
UPP1
LOC112443007
C3H1orf162
CYTIP
ADORA2A
CD300A
BTG2
UBE2W
LOC100847554
SELL
IL18R1
ZNF263
RC3H2
ABCA6
CCR1
ARID4B
GAB1
FAM234A
LOC107133053
MORC3
USP15
SLC16A6
LOC101906317
LOC100847818
HIP1R
USP32
SLC25A19
MIDN
RELB
MYBPH
LOC527744
MIER1
VAT1L
RASA2
RUFY4
UBR1
EMB
TCN1
WDR33
IFITM3_1
TAPBPL
NABP1
ZNF614
PAPD4
LY96
NAIP
MIER3
LOC112445973
MOB1A
AOAH
SAT1
BRWD3
LOC107133088
UBAP1
PSPC1
LOC783680
PLB1
IPMK
UBE2B
IFNAR2
KLHL8
CDS2
PTBP2
FAM129A
CWC22
CCRL2
LOC112445930
JAK2
LOC534155
DDX3X
ISG20
FNBP4
THBD
IGSF6
ISG15
RNPEP
LOC112443413
TG
XCR1
LITAF
TTC33
BASP1
LOC104969531
LOC618409
AMPD3
LOC112442414
BCLAF3
PGLYRP4
DEDD2
CD53
FGFR1OP2
FGR
LOC107131590
WLS
ELL
CRISPLD2
LOC112443133
PSTPIP2
VCPIP1
LST1
SLA
S100A9
USP3
LOC617565
NFKB1
CCNJL
TMEM156
GRB2
ECE1
TCIRG1
CATHL3
KPNA4
LOC112448777
AFF4
TRIM56
DIXDC1
CYLD
LOC107132519
LOC104975781
LOC104973105
ERO1B
VSIR
LOC101903165
LOC112449349
ATAD2B
SERPINB4
BATF3
MTMR9
DAGLB
LOC112449617
LOC104974562
LOC101904383
KMO
SLC22A1
SP3
RNF149
GOSR1
CHD1
USP34
DENND3
C5AR2
TMEM154
TNFRSF1A
FITM1
HRH2
XRN1
CPB2
LOC112442262
C25H7orf50
MB21D2
PSMC3IP
SF3B1
RUBCNL
NCF1
LOC112449306
ST7L
RASGRP4
LRRK2
BTG1
LOC101901948
WASHC4
LOC107131803
LOC107132783
LOC104973829
LOC112448847
LOC100848752
LOC104970598
SWT1
AFF2
SOD2
CCNL2
STEAP4
IFITM1
LOC104971364
BCL6
LOC112443195
PTAFR
LOC107132901
LOC107133110
USF1
LOC112447080
CNOT6L
ZNF292
PAPOLG
IL18RAP
XIAP
STAU1
PNRC1
IL2RA
LOC104973826
OVGP1
KMT5B
IMPG2
MXD1
LOC107133075
SDSL
PIK3R6
ADGRG3
LOC112446780
STAT3
MARCKS
NLGN3
LOC786348
DDIT4
LOC101904248
PELI1
LOC407171
PXN
LOC112444774
LOC104976076
HIC1
SP4
CCDC112
ANKRD12
CREBRF
BIRC3
FMNL1
LOC107131942
SUSD6
OMG
NKTR
UPF2
SNAPC1
GPR84
MAP3K2
KMT2E
CHST11
DMXL2
AGBL3
CATHL1
LOC508666
TIAM2
RIPOR2
ADAM19
LOC507247
LOC617503
BRF2
KDM7A
RASSF2
FBXL5
LOC104970103
LOC112448782
MMP25
SLC6A9
MYLIP
TMF1
ACSL4
JUNB
FBRS
SNN
CXCR2
HCK
IQSEC1
ZNF148
TDRD10
PIAS1
GPR4
TANK

[2B]

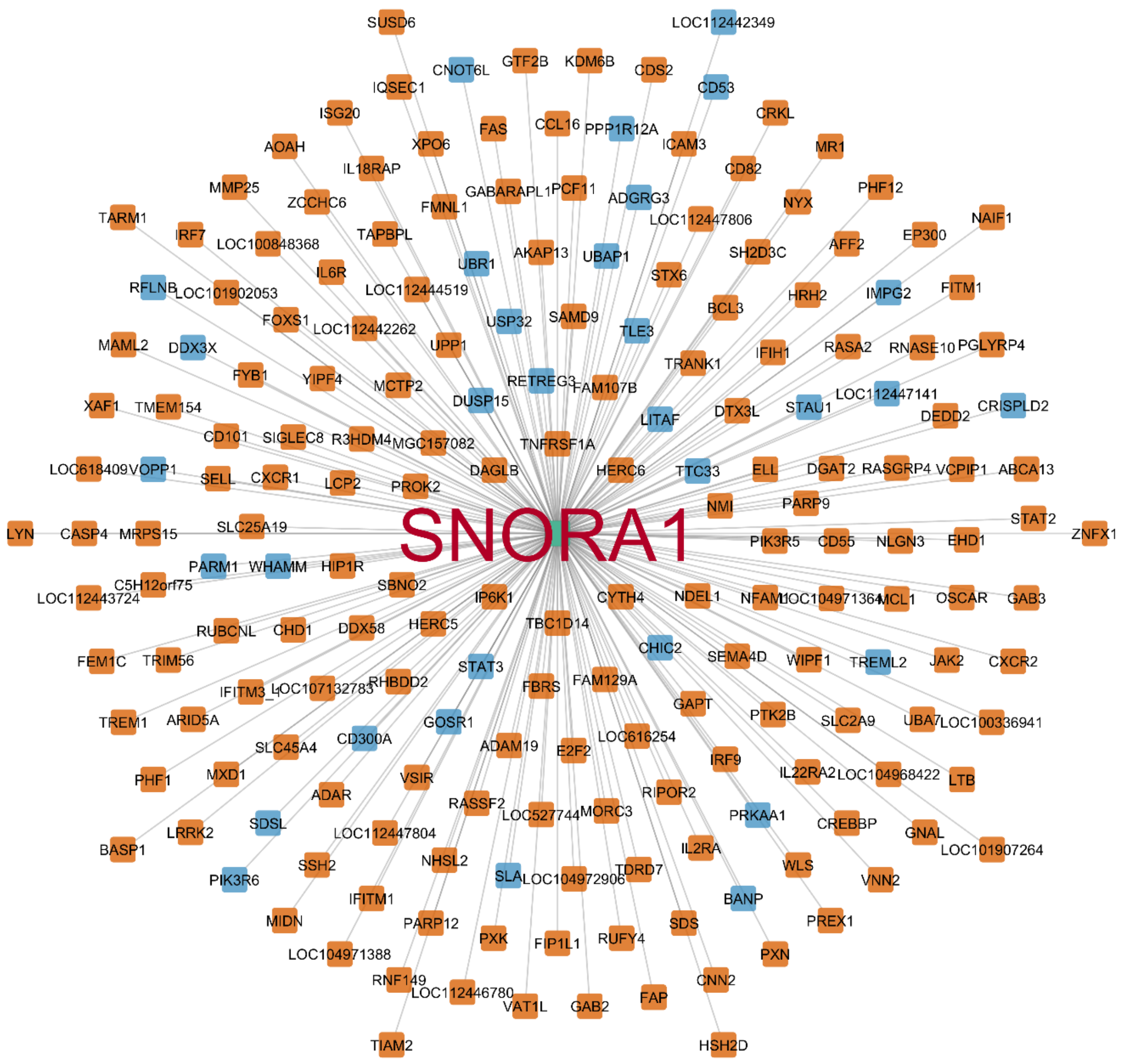
SNORA1
SUSD6
LOC112442349
CNOT6L
GTF2B
KDM6B
CDS2
IQSEC1
CD53
ISG20
FAS
CCL16
PPP1R12A
CRKL
AOAH
XPO6
ICAM3
MR1
IL18RAP
CD82
MMP25
GABARAPL1
PCF11
ADGRG3
NYX
PHF12
ZCCHC6
FMNL1
LOC112447806
TARM1
NAIF1
IRF7
TAPBPL
AFF2
EP300
LOC100848368
AKAP13
UBAP1
SH2D3C
UBR1
IL6R
STX6
RFLNB
LOC101902053
LOC112444519
IMPG2
FITM1
HRH2
FOXS1
SAMD9
BCL3
USP32
LOC112442262
TLE3
MAML2
DDX3X
UPP1
IFIH1
RASA2
RNASE10
PGLYRP4
FYB1
TRANK1
YIPF4
MCTP2
RETREG3
FAM107B
LOC112447141
XAF1
TMEM154
DUSP15
STAU1
CRISPLD2
DTX3L
LITAF
DEDD2
CD101
SIGLEC8
R3HDM4
MGC157082
TNFRSF1A
LOC618409
VOPP1
DAGLB
HERC6
TTC33
ELL
DGAT2
RASGRP4
VCPIP1
ABCA13
SELL
CXCR1
LCP2
PROK2
NMI
PARP9
LYN
CASP4
MRPS15
SLC25A19
STAT2
ZNFX1
PIK3R5
CD55
NLGN3
EHD1
PARM1
WHAMM
HIP1R
C5H12orf75
SBNO2
LOC112443724
IP6K1
CYTH4
NDEL1
NFAM1
LOC104971364
MCL1
OSCAR
GAB3
RUBCNL
CHD1
DDX58
HERC5
TBC1D14
CHIC2
FEM1C
TRIM56
SEMA4D
WIPF1
TREML2
JAK2
CXCR2
STAT3
IFITM3
LOC107132783
RHBDD2
FBRS
FAM129A
GAPT
TREM1
ARID5A
PTK2B
SLC2A9
UBA7
LOC100336941
GOSR1
CD300A
LOC616254
SLC45A4
ADAM19
E2F2
IRF9
PHF1
MXD1
VSIR
IL22RA2
LOC104968422
LTB
ADAR
RIPOR2
RASSF2
LOC527744
MORC3
PRKAA1
SDSL
CREBBP
GNAL
LRRK2
LOC112447804
IL2RA
BASP1
LOC101907264
NHSL2
SSH2
TDRD7
SLA
LOC104972906
WLS
PIK3R6
VNN2
IFITM1
BANP
MIDN
PARP12
SDS
PREX1
PXK
FIP1L1
RUFY4
LOC104971388
PXN
RNF149
LOC112446780
CNN2
VAT1L
GAB2
FAP
TIAM2
HSH2D

Figure S2. Correlation network between hub snoRNAs and target DE genes for Staphylococcus aureus versus the control. (A) SNORA66 and the DE target genes. B) SNORA1 and the DE target genes. The orange nodes represent downregulated DE genes and the blue nodes represents upregulated DE genes.

[3A]

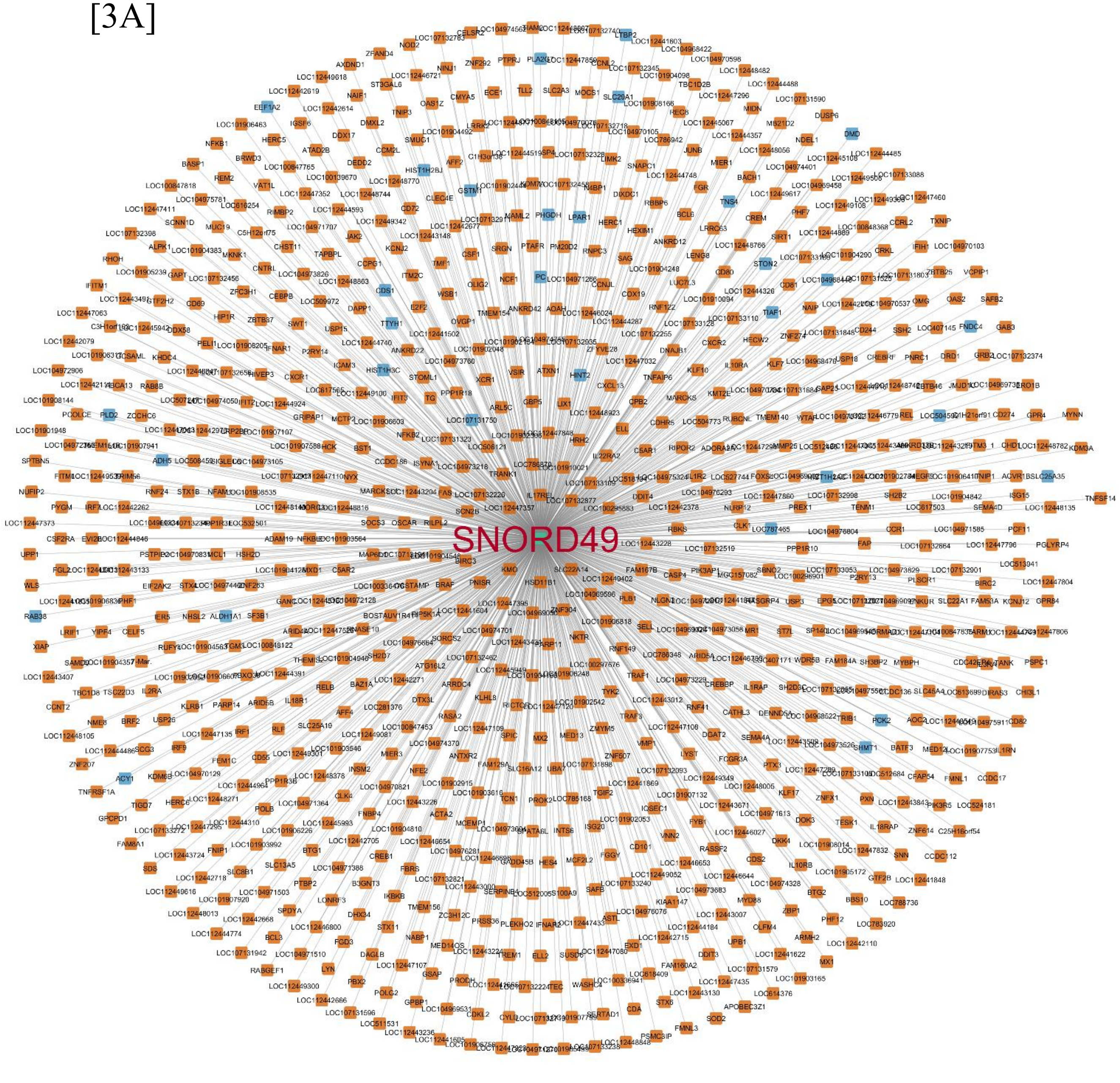

[3B]

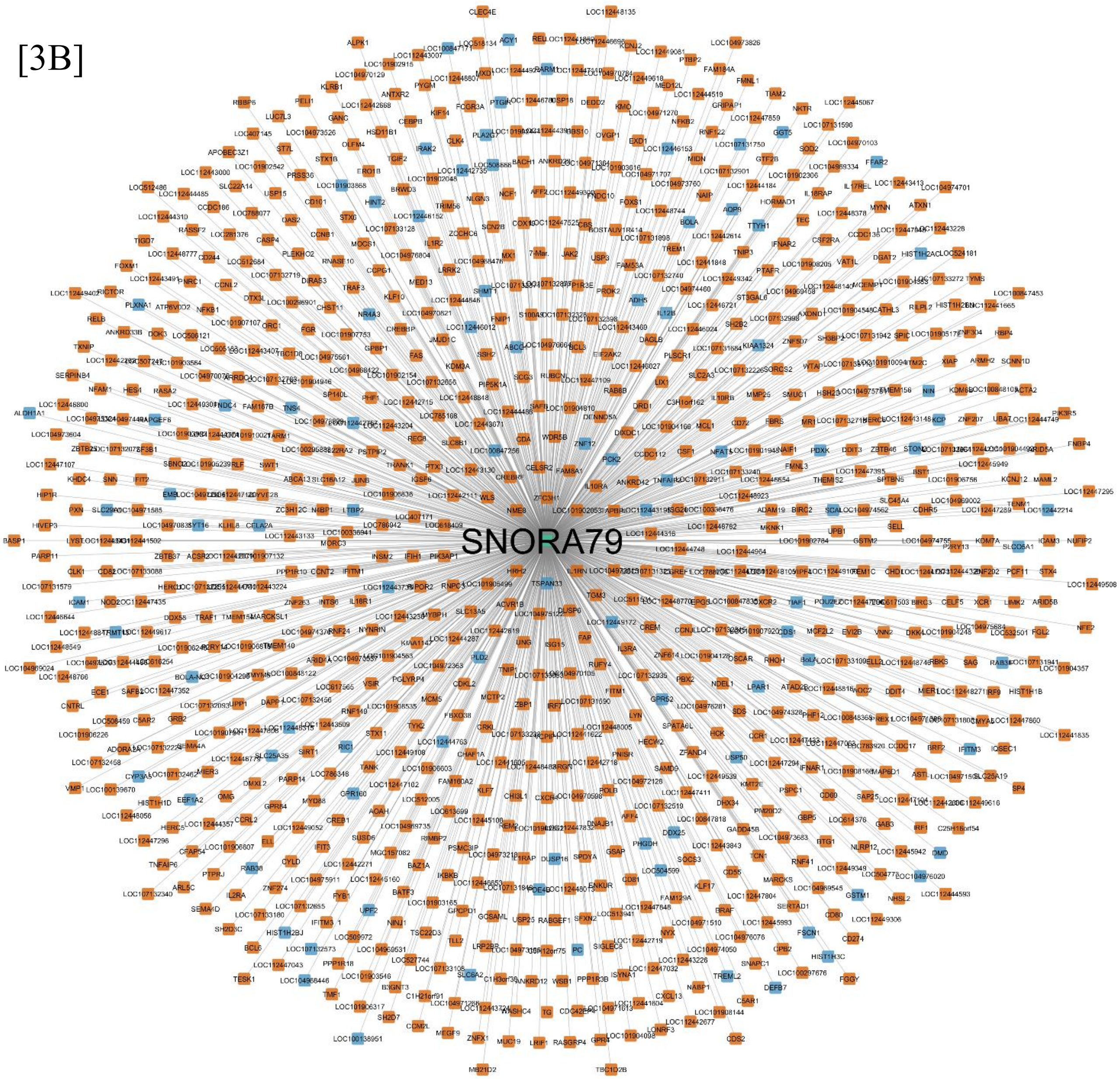

Figure S3. Correlation network between hub snoRNAs and target DE genes for *Staphylococcus chromogenes* versus the control. (A) SNORD49 and the DE target genes. B) SNORA79 and the DE target genes. The orange nodes represent downregulated DE genes and the blue nodes represents upregulated DE genes.
